# GPT-4o and the quest for machine learning interpretability in ICU risk of death prediction

**DOI:** 10.1186/s12911-025-03224-z

**Published:** 2025-10-13

**Authors:** Moein E. Samadi, Kateryna Nikulina, Sebastian Johannes Fritsch, Andreas Schuppert

**Affiliations:** 1 Institute for Computational Biomedicine, RWTH Aachen University, Aachen, Germany; 2 Center for Computational Life Sciences, RWTH Aachen University, Aachen, Germany; 3 Department of Intensive Care Medicine, University Hospital RWTH Aachen, Aachen, Germany; 4 Jülich Supercomputing Centre, Forschungszentrum Jülich, Jülich, Germany; 5 Center for Advanced Simulation and Analytics (CASA), Forschungszentrum Jülich, Jülich, Germany

**Keywords:** Interpretable machine learning, Large language models, Hybrid mechanistic/data-driven modeling, Feature clustering, ICU risk of death prediction

## Abstract

**Background:**

Clinical utilization of machine learning is hampered by the lack of interpretability inherent in most non-linear black box modeling approaches, reducing trust among clinicians and regulators. Advanced large language models offer a potential framework for integrating medical knowledge into these models, potentially enhancing their interpretability.

**Methods:**

A hybrid mechanistic/data-driven modeling framework is presented for developing an ICU risk of death prediction model for mechanically ventilated patients. In the mechanistic modeling part, GPT-4o is used to generate detailed medical feature descriptions, which are then aggregated into a comprehensive corpus and processed with TF-I DF vectorization. Fuzzy C-means clustering is subsequently applied to these vectorized features to identify significant mortality cause-specific feature clusters, and a physician reviewed the resulting clusters to validate their relevance to actionable insights for clinical decision support. In the data-driven part, the identified clusters inform the creation of XGBoost-based weak classifiers, whose outcomes are combined into a single XGBoost-based strong classifier through a hierarchically structured feed-forward network. This process results in a novel GPT hybrid model for ICU risk of death prediction.

**Results:**

This study enrolled 16,018 mechanically ventilated ICU patients, divided into derivation (12,758) and validation (3,260) cohorts, to develop and evaluate a GPT hybrid model for predicting in-ICU death. Leveraging GPT-4o, we implemented an automated process for clustering mortality cause-specific features, resulting in six feature clusters: Liver Failure, Infection, Renal Failure, Hypoxia, Cardiac Failure, and Mechanical Ventilation. This approach significantly improved upon previous manual methods, automating the reconstruction of structured hybrid models. While the GPT hybrid model showed similar predictive accuracy to a Global XGBoost model, it demonstrated superior interpretability and clinical relevance by incorporating a wider array of features and providing a hierarchical structure of feature importance aligned with medical knowledge.

**Conclusion:**

We introduce a novel approach to predicting in-ICU risk of death for mechanically ventilated patients using a GPT hybrid model. Our methodology demonstrates the potential of integrating large language models with traditional machine learning techniques to create interpretable and clinically relevant predictive models.

**Supplementary information:**

The online version contains supplementary material available at 10.1186/s12911-025-03224-z.

## Background

Clinical utilization of machine learning demonstrates promising capabilities in predictive modeling; however, substantial challenges arise when implementing these models in practice  [1–3]. One major issue is the lack of interpretability inherent in most non-linear black box modeling approaches, which makes it difficult for clinicians to trust and understand the reasoning behind the predictions  [4–7].

Many current approaches, while promising, fall short of providing the level of transparency required for widespread clinical adoption  [8]. For instance, Bayesian inference and causality learning can offer insights into the relationships between variables  [9–11], but they often require extensive domain expertise to interpret correctly. Kolmogorov-Arnold Networks  [12–14], though powerful in their ability to approximate complex functions, may still produce results that are not easily understandable to clinicians without a background in advanced mathematics.

Hybrid mechanistic/data-driven modeling, which aims to integrate data-driven predictions with available mechanistic knowledge, represents a promising approach  [15, 16]. However, systematically incorporating medical expertise into the learning strategies of data-driven models is challenging and often case-specific  [17, 18]. The main issue lies in bridging the gap between the intrinsic nature of medical knowledge, which frequently describes mechanisms and underlying principles, and data-driven learning strategies that focus on pattern recognition and probabilistic associations. Prior structured hybrid modeling approaches, particularly those used for intensive care unit (ICU) risk of death prediction, often require extensive manual input from medical experts to reconstruct structural networks. This manual process can be both time-consuming and prone to error, typically involving multiple rounds of refinement by an informatician followed by further review by a medical expert  [17, 18].

One promising approach to bridge this gap is the utilization of large language models (LLMs)  [19]. LLMs, such as Generative Pre-trained Transformer (GPT) models represented by OpenAI’s ChatGPT, have demonstrated an impressive ability to process and generate human-like text  [20]. Trained on vast amounts of data, LLMs have initiated a significant shift in artificial intelligence applications for healthcare  [21, 22], prompting the need for ethical and secure pipelines  [23, 24]. These models have demonstrated remarkable potential in various clinical studies, including tasks such as medical text summarization  [25], diagnosis of complex clinical cases  [26], extracting structured data from clinical notes  [27], and clinical decision support  [28].

Of particular interest is the potential of advanced LLMs, such as GPT-4o, to generate guided data for structural clinical knowledge representation  [29]. This approach offers a promising framework for integrating medical knowledge into the learning process, potentially enhancing the interpretability of machine learning-based clinical predictive models, which forms the focus of this study.

This paper presents a hybrid mechanistic/data-driven modeling framework for developing an ICU risk of death prediction model for mechanically ventilated patients. The mechanistic modeling part of our approach leverages the capabilities of GPT-4o to generate medical feature descriptions. These descriptions are then processed and clustered to identify distinct mortality-related patterns. In the data-driven part of our approach, we use these clusters to construct and train XGBoost-based  [30] weak classifiers that act on each feature cluster, and their outcomes are subsequently combined into a single XGBoost-based strong classifier through a hierarchically structured feed-forward network. This process results in a novel *GPT hybrid model* for ICU risk of death prediction.

In contrast to prior interpretable machine learning studies in healthcare that often rely on post-hoc explanation methods or highly specialized domain knowledge rules  [31, 32], our approach systematically transforms LLM-generated medical feature descriptions into distinct, human-understandable sub-models that drive a hierarchically structured feed-forward network. By focusing on cause-specific sub-models, this design mitigates uncertainties often arising from global feature effects and concentrates explainability on the sub-models. Furthermore, leveraging GPT-4o in this manner reduces the need for extensive manual network construction by clinical experts, offering a more automated yet clinically coherent framework for hybrid modeling.

Moreover, hybrid models are frequently used to enhance extrapolation properties  [16], boost predictive performance  [33], mitigate overfitting  [34], and identify an optimal design of an underlying process  [35]. Our work extends these benefits by demonstrating a proof of concept for enhanced interpretability of hybrid models in healthcare.

We evaluate our GPT hybrid model using a large cohort of mechanically ventilated ICU patients, comparing its performance and interpretability to a *Global XGBoost model*, which was trained on features without any pre-structuring. Through this work, we aim to demonstrate how integrating medical knowledge via large language models can automate the network reconstruction process for hybrid modeling and enhance the interpretability of clinical predictive models without compromising their accuracy, potentially increasing clinician trust and adoption in critical care settings.

## Methods

### Data source and population

For this study, we utilized data from over 16,600 admissions to the ICU at University Hospital RWTH Aachen. These data were collected as part of the project titled “Algorithmic Surveillance of ICU Patients with Acute Respiratory Distress Syndrome” (ASIC)  [36]. The ASIC project was an integral component of the SMITH consortium  [37], which operated under the German Medical Informatics Initiative. This study is reported in accordance with the TRIPOD-AI guideline for reporting AI-based prediction model studies  [38].

The ASIC Calibration database was retrospectively sourced and thoroughly depersonalized from ICU patients involved in the ASIC project, consisting of data from 13,067 ICU admissions within the time frame from 2009 to 2020. We used this database, referred to as *the derivation cohort*, after applying our study’s inclusion and exclusion criteria, to develop risk of death risk prediction models. The ASIC Control database, consisting of data from 3591 ICU admissions, was retrospectively sourced and thoroughly depersonalized within the time frame from 2020 to 2021, from ICU patients involved in the ASIC project. After applying our study’s inclusion and exclusion criteria, we used the ASIC Control database as *the validation cohort*. Using the hospital’s pseudonymised patient index, we ensured that each individual appears in one cohort only. If a patient had admissions in both periods, all of that patient’s data were assigned to the derivation cohort.

**Fig. 1:**
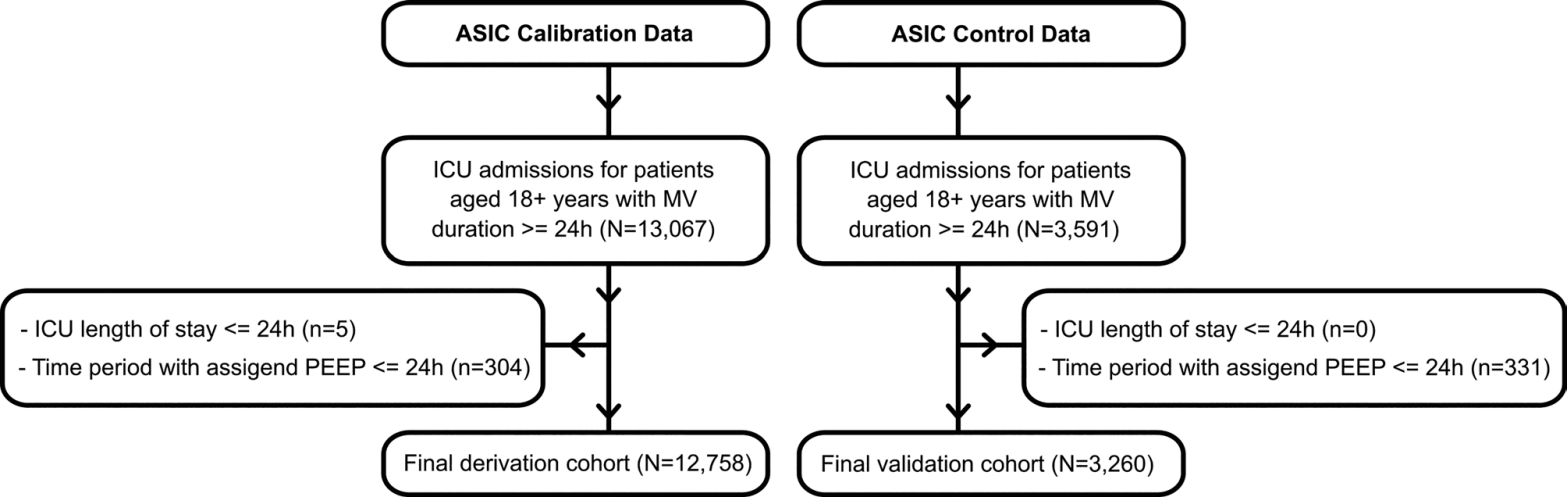
Consort diagram of derivation and validation cohorts

In this study, we included all patients aged 18 years or older who underwent invasive mechanical ventilation (MV) for a cumulative duration of at least 24 hours. The exclusion criteria were as follows: firstly, we removed all patients who died during their first day at the ICU. Next, we calculated the first 24-hour stay at the ICU for a patient starting from the time point when they received MV. The first available value of positive end-expiratory pressure (PEEP) was used to define MV. Patients who were ventilated for less than 24 hours starting from the first PEEP value were excluded from further analysis. The consort diagram illustrating the selection of the study cohort is presented in Fig. 1.

### Feature extraction

We collected data on patient demographics, comorbidities, vital signs, laboratory values, and criteria-based flags. The mean, median, minimum, maximum, and standard deviation of vital signs and laboratory values were extracted during the first 24 hours of MV, which is defined as *the observation window*. The extracted features and their categorizations are summarized in Table 1.

**Table 1:** Summary of extracted features

Category	Feature	Units/Notes
Demographics	Age	Years
	Gender	Male/Female
	BMI category	Categories: 0 - underweight, 1 - normal, 2 - overweight, 3 - stage 1 obesity, 4 - stage 2 obesity, 5 - morbidly obese
Comorbidities	Chronic heart failure	Binary (Yes/No)
	Chronic renal failure	Binary
	Diabetes	Binary
	Hypertension	Binary
	Immune suppression	Binary
	Obesity	Binary
	Thoracic trauma	Binary
Vital Signs	Central venous pressure (CVP)	mmHg
	Diastolic blood pressure	mmHg
	Fraction of inspired oxygen (FiO_2_)	%
	Heart rate	Beats per minute
	Lung compliance	mL/cmH_2_O
	Mean arterial pressure (MAP)	mmHg
	Partial pressure of arterial oxygen to fraction of inspired oxygen ratio (PaO_2_/FiO_2_)	mmHg
	Positive end-expiratory pressure (PEEP)	cmH_2_O
	Respiratory rate	Breaths per minute
	Peripheral oxygen saturation (SpO_2_)	%
	Systolic blood pressure	mmHg
	Temperature	^∘^C
Laboratory Values	Albumin	umol/L
	Alanine aminotransferase (ALT)	U/L
	Aspartate transaminase (AST)	U/L
	Bicarbonate	mmol/L
	Bilirubin	umol/L
	B-type natriuretic peptide (BNP)	pmol/L
	Blood urea nitrogen (BUN)	mmol/L
	BUN/albumin ratio	Dimensionless
	BUN/creatinine ratio	Dimensionless
	Creatine kinase (CK)	U/L
	C-reactive protein (CRP)	nmol/L
	Creatinine	umol/L
	Estimated glomerular filtration rate (eGFR)	mL/min/1.73 m^2^ - Calculated using the CKD-EPI formula, excluding the consideration of race [39].
	Hematocrit	%
	Hemoglobin	mmol/L
	Interleukin 6 (IL-6)	pg/mL
	International normalized ratio (INR)	Dimensionless
	Lactate	mmol/L
	Lactate dehydrogenase (LDH)	U/L
	Platelet count	10^3^/uL
	Procalcitonin (PCT)	ng/mL
	Partial pressure of oxygen (PO_2_)	mmHg
	Partial thromboplastin time (pTT)	Seconds
	White blood cell count (WBC)	10^3^/uL
Criteria-based Flags	Acute Respiratory Distress Syndrome (ARDS) flag	Binary - Defined as a PaO_2_/FiO_2_ ratio of 300 or less and a PEEP of 5 cmH2O or higher sustained for at least 8 consecutive hours [40].
	Organ dysfunction flag	Binary - Defined as a platelet count less than $100 \times 10^9/L$, a lactic acid level greater than 2 mmol/L, or an INR exceeding 1.5 [41].

Demographic information included age, gender, and body mass index (BMI), which was categorized into six classes for anonymization purposes. Comorbidities considered were various chronic conditions and trauma.

Vital signs encompass physiological measurements relevant to MV and patient monitoring, such as blood pressures, heart rate, oxygenation indices, and others. Laboratory values included a comprehensive set of biochemical parameters, hematological indices, and markers of organ function and inflammation.

Criteria-based flags were defined to identify the presence of Acute Respiratory Distress Syndrome (ARDS) and organ dysfunction based on specific clinical criteria. The ARDS flag was determined using the PaO_2_/FiO_2_ ratio and PEEP values, while the organ dysfunction flag was based on platelet count, lactic acid levels, and INR values.

### Study objective and clinical outcome

The objective of this study was to develop a structural knowledge-representation framework using LLMs that yields interpretable predictions of ICU mortality. The clinical outcome modeled was in-ICU death within the first seven days after initiation of mechanical ventilation.

### TF-IDF membership

TF-IDF, or Term Frequency-Inverse Document Frequency, is a statistical measure used to evaluate the importance of a word in a document relative to a collection of documents (corpus)  [42, 43]. It combines two key metrics: term frequency (TF), which measures how often a term appears in a document, and inverse document frequency (IDF), which measures how unique a term is across the entire corpus by penalizing common terms. By multiplying TF and IDF, the TF-IDF score of a term increases with the number of times the term appears in a document, but is offset by how frequently it appears in other documents.

The TF-IDF scores of all terms in a document generate a numerical representation (vector) that highlights the document’s unique content features. The vectors of all documents in a corpus can then be employed in clustering algorithms to group documents based on content similarity.

### Fuzzy C-means clustering

Fuzzy C-means clustering is a soft clustering technique that allows each data point to belong to multiple clusters with varying membership degrees, unlike traditional k-means, which assigns points to a single cluster  [44]. Fuzzy C-means uses a membership function to assign each data point a membership degree between 0 and 1. The algorithm minimizes an objective function that balances membership degrees and distances from cluster centers, iteratively adjusting both until convergence.

Fuzzy C-means clustering was applied to a TF-IDF matrix derived exclusively from GPT-generated feature descriptions. In this matrix, the rows correspond to the N individual medical features, and the columns correspond to the semantic dimensions captured by the TF-IDF representation. The fuzziness exponent (m) and the number of clusters (K) were set to 1.2 and 6, respectively; these values were chosen empirically to balance cluster overlap and separation. Fuzzy C-means clustering produced a k × N membership matrix U, whose element ${u_{ij}}$ quantifies the degree to which feature j belongs to cluster i. Each feature was assigned to the cluster with the highest membership, while ties were retained so that features with comparable memberships could contribute to multiple clusters. Importantly, only the semantic TF-IDF vectors informed this step; no numerical patient-level measurements were included. After the clusters were finalized and clinician-labeled (e.g., “Liver failure”, “Hypoxia”), the corresponding numeric variables for each cluster were extracted from the derivation cohort and passed into an XGBoost weak learner trained exclusively on that cluster’s variables.

### Model development

**Fig. 2:**
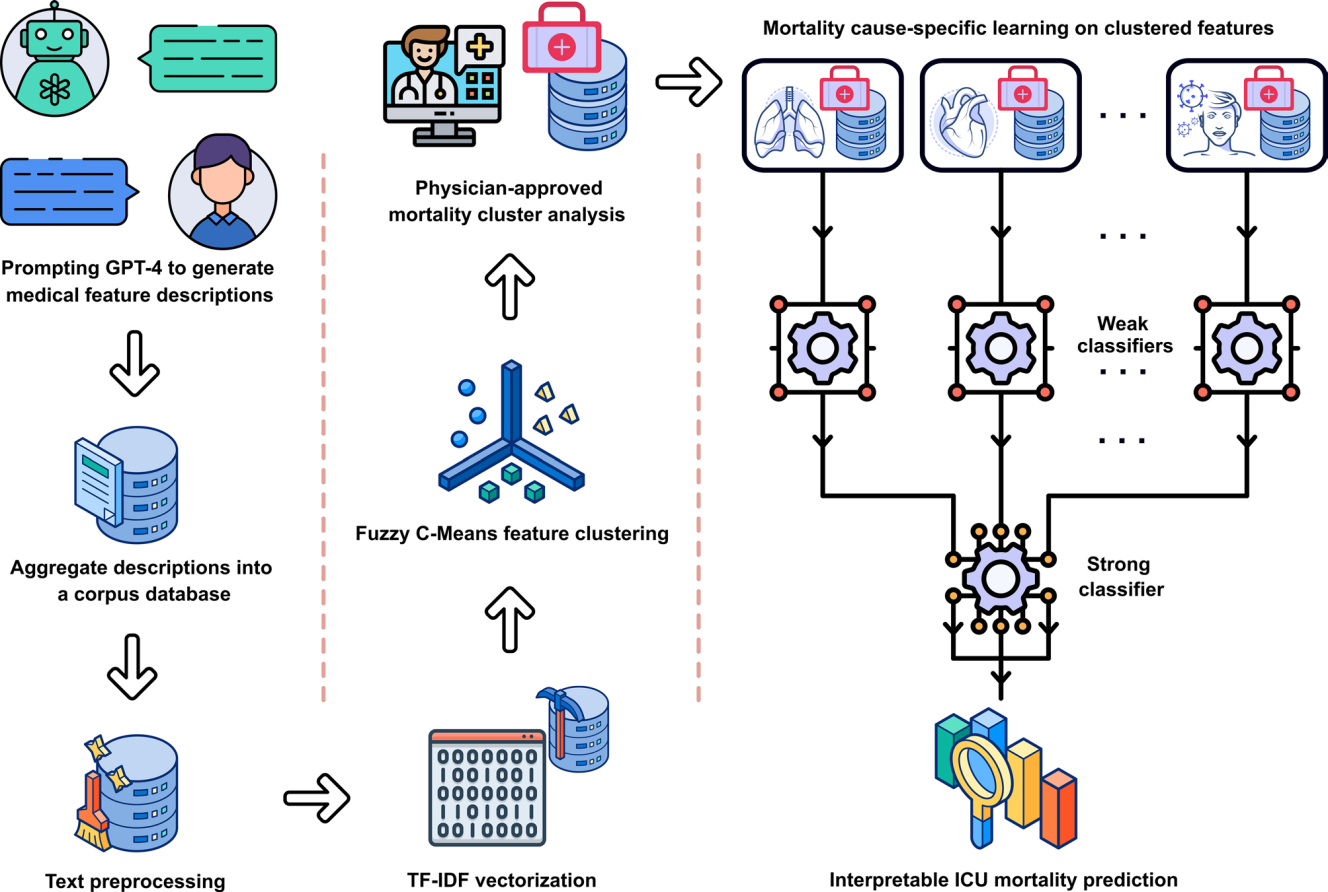
The overall model development workflow

The overall model development workflow is presented in Fig. 2. Our development of an interpretable ICU risk of death prediction model begins with generating medical feature descriptions using GPT-4o, which provides detailed information on the impact of each extracted feature listed in Table 2 on ICU death rates for mechanically ventilated patients. These AI-generated descriptions are aggregated into a comprehensive corpus database, forming the foundational dataset for structural knowledge representation.

**Table 2:** Clinical characteristics of derivation and validation cohorts

	Derivation (N = 12,758)	Validation (N = 3,260)	P value
**Demographics**			
Age${}^{{\rm a}}$	65 (20)	58.2 (16.8)	< 0.05
BMI category${}^{{\rm a}}$	2 (2)	2 (2)	< 0.05
Gender (male)${}^{{\rm b}}$	8325 (65.3)	2163 (66.3)	0.24
**Comorbidities and Flags**			
Chronic heart failure${}^{{\rm b}}$	7500 (58.8)	1796 (55.1)	< 0.05
Chronic renal failure${}^{{\rm b}}$	2241 (17.6)	657 (20.2)	< 0.05
Diabetes${}^{{\rm b}}$	3516 (27.6)	1090 (33.4)	< 0.05
Hypertension${}^{{\rm b}}$	7712 (60.4)	2084 (63.9)	< 0.05
Immune suppression${}^{{\rm b}}$	1244 (9.8)	425 (13)	< 0.05
Obesity${}^{{\rm b}}$	3410 (26.7)	1013 (31.1)	< 0.05
Thoracic trauma${}^{{\rm b}}$	682 (5.3)	274 (8.4)	< 0.05
ARDS${}^{{\rm b}}$	8318 (65.2)	2238 (68.7)	< 0.05
Organ dysfunction${}^{{\rm b}}$	292 (2.3)	89 (2.7)	0.14
**Vital Signs**			
CVP${}^{{\rm c}}$	12.09 (6.73)	11.68 (6.71)	< 0.05
Diastolic blood pressure${}^{{\rm c}}$	59.15 (8.41)	58.22 (8.54)	< 0.05
FiO$_2{}^{{\rm c}}$	45.1 (11.88)	44.58 (12.75)	< 0.05
Heart rate${}^{{\rm c}}$	84.97 (16.56)	84.53 (16.36)	0.178
Lung compliance${}^{{\rm c}}$	62.65 (35.15)	57.97 (32.54)	< 0.05
MAP${}^{{\rm c}}$	78.33 (8.73)	76.86 (8.62)	< 0.05
PaO_2_/FiO$_2{}^{{\rm c}}$	270.61 (100.67)	255.82 (98.84)	< 0.05
PEEP${}^{{\rm a}}$	7.14 (2.62)	7.14 (2.78)	0.976
Respiratory rate${}^{{\rm c}}$	17.68 (3.91)	17.91 (4.34)	< 0.05
SpO$_2{}^{{\rm a}}$	98.11 (2.72)	97.21 (3.15)	< 0.05
Systolic blood pressure${}^{{\rm c}}$	119.33 (14)	117.56 (13.86)	< 0.05
Temperature${}^{{\rm c}}$	36.78 (0.97)	36.94 (0.79)	< 0.05
**Laboratory Values**			
Albumin${}^{{\rm c}}$	396.53 (196.08)	618.73 (2971.28)	< 0.05
ALT${}^{{\rm a}}$	27 (34)	29 (41.5)	< 0.05
AST${}^{{\rm a}}$	48 (67.5)	48 (70.33)	0.573
Bicarbonate${}^{{\rm c}}$	23.62 (3.72)	25.16 (4.01)	< 0.05
Bilirubin${}^{{\rm a}}$	11.03 (11.8)	10.69 (11.66)	< 0.05
BNP${}^{{\rm a}}$	1689.5 (5314.1)	1858 (5287.25)	0.718
BUN${}^{{\rm a}}$	7.18 (5.34)	7.26 (6.07)	0.186
BUN/Albumin ratio${}^{{\rm a}}$	0.02 (0.02)	0.02 (0.02)	0.32
BUN/Creatinine ratio${}^{{\rm c}}$	7.52 (3.74)	7.6 (3.96)	0.243
CK${}^{{\rm a}}$	185.83 (331)	145 (250.6)	< 0.05
CRP${}^{{\rm a}}$	609.52 (1162.86)	676.9 (1212.98)	< 0.05
Creatinine${}^{{\rm a}}$	1.04 (0.73)	1.05 (0.81)	0.487
eGFR${}^{{\rm a}}$	67.45 (47.53)	67.35 (51.04)	0.48
Hematocrit${}^{{\rm a}}$	29.92 (6.5)	30.71 (7.56)	< 0.05
Hemoglobin${}^{{\rm a}}$	6.06 (1.34)	6.25 (1.56)	< 0.05
IL-6${}^{{\rm a}}$	346.4 (1090.7)	248.4 (604.89)	< 0.05
INR${}^{{\rm a}}$	1.17 (0.24)	1.2 (0.26)	< 0.05
Lactate${}^{{\rm a}}$	1.5 (1.58)	1.35 (1.42)	< 0.05
LDH${}^{{\rm a}}$	282.5 (202)	305 (238)	< 0.05
PCT${}^{{\rm a}}$	0.7 (3)	0.69 (2.92)	0.808
Platelet${}^{{\rm c}}$	202.34 (107.53)	206.03 (108.32)	0.082
PO$_2{}^{{\rm c}}$	107.79 (24.76)	99.4 (22.44)	< 0.05
pTT${}^{{\rm a}}$	30.9 (8.57)	31.25 (9.74)	0.268
WBC${}^{{\rm c}}$	12.8 (7.77)	13.29 (11.92)	< 0.05

^a^ Presented as median (interquartile range) and analyzed using the Mann–Whitney U test

^b^ Presented as frequencies (percentages) and analyzed using the Chi-square test

^c^ Presented as mean (standard deviation) and analyzed using the Student’s t-test

We employed a structured prompt design to generate medically relevant and concise feature descriptions. As detailed in Additional file 1, the general structure of our prompts is as follows:

*Answer this question in [Word Count] words:*

*How does [Medical Feature] impact the ICU mortality rates of mechanically ventilated patients?*

Three key elements define this prompt design. First, it sets a specific word count to ensure responses remain concise yet informative. Second, by focusing on a single medical feature, the prompt allows for an in-depth examination of that feature’s impact. Finally, it specifies a target population of mechanically ventilated patients, tailoring the analysis to this particular study group. This approach generates a detailed description of each feature’s influence on ICU death rates for mechanically ventilated patients.

After generating the initial descriptions, we reviewed them to detect inconsistencies and identify irrelevant or redundant information. We also cross-checked suspicious or ambiguous statements. If discrepancies were found, we repeated the query and validated the results until the final text was clinically and contextually sound. The complete cross-checked corpus database is available in Additional file 1.

Next, we employed a multi-step text preprocessing pipeline to prepare the corpus database for computational analysis. First, we cleaned and standardized the text, removing punctuation, symbols, and stop words that did not contribute to the semantic content. Then, we applied tokenization to split the text into analyzable units, followed by stemming and lowercasing to reduce vocabulary dimensionality. Finally, we vectorized the processed text using the TF-IDF technique, transforming the textual data into numerical representations suitable for computational analysis.

We then applied fuzzy C-means clustering to the vectorized data to identify significant mortality cause-specific feature clusters. Subsequently, a physician reviewed the resulting clusters, validating their relevance to specific mortality causes in mechanically ventilated ICU patients and assigning appropriate cluster names.

For each identified feature cluster, we trained weak classifiers using the derivation data, focusing on the unique characteristics of each cluster. The outcomes of these weak classifiers were then combined and boosted through a strong classifier  [45, 46], resulting in a GPT hybrid model for ICU risk of death prediction. We used XGBoost classifiers for both the weak and strong classifiers.

Running the entire workflow on a CPU-only workstation with an Intel(R) Core(TM) CPU (i7-8565 U @ 1.80 GHz) and 16 GB RAM, from LLM prompting through text pre-processing, TF-IDF vectorization, fuzzy C-means clustering, and grid-search hyper-parameter tuning with stratified 5-fold cross-validation  [47], can be finished in well under two hours, with peak memory less than 9 GB. This shows the workflow is practical on routine hospital hardware.

### Global XGBoost model

To benchmark the GPT hybrid approach, we trained a “global” XGBoost classifier on the complete feature list given in Table 1, i.e., without any pre-structuring or cluster information. We used the Python XGBoost library (v2.1.6) with the binary logistic objective and the Area Under the Receiver Operating Characteristic Curve (AUC-ROC) as the optimization metric. Hyperparameters were selected by grid-search with stratified 5-fold cross-validation on the derivation cohort, and Early stopping (10 rounds) was implemented to prevent overfitting, identical to the procedure used for the GPT hybrid workflow. After tuning, the model was re-trained on the full derivation cohort with the optimal hyperparameters and subsequently evaluated on the independent validation cohort.

### Feature importance extraction

To identify which features and feature clusters most strongly affected our GPT hybrid model’s predictive performance, we employed the XGBoost Python implementation’s built-in feature-importance functionality. This calculates how often and how significantly each feature is used to split decision trees in the boosted ensemble. Specifically, we extracted the “gain” metric, indicating the improvement in the model’s objective function (logistic loss for risk of death prediction) each time a feature is used for splitting. Conceptually, this approach aligns with principles like Gini impurity or information gain in traditional decision trees: features that reduce impurity (or improve model loss) the most over many splits receive the highest importance scores.

We aggregated these gain values from all trees to compute a single importance score per feature. Each feature cluster’s overall importance is determined by the XGBoost-based strong classifier’s learned weight, while each feature’s relative importance within its cluster comes from that cluster’s XGBoost-based weak classifier. Mathematically, if *w*_*i*_ is the learned weight for cluster *i* and *f*_*ij*_ is the importance of feature *j* in cluster *i*, then the final importance of feature *j* in cluster *i* is $w_i \times f_{ij}$. These importances are normalized so that they sum to 1 across clusters. This hierarchical approach is analogous to grouping features by major clinical mortality causes and then assigning importance within each group, thereby clarifying how each cluster and feature contributes to the final risk of death prediction.

### Statistical analysis

Continuous variables were presented as mean (standard deviation) for normally distributed data or as median (interquartile range) for non-normally distributed data. Categorical variables were reported as frequencies (percentages). To compare clinical characteristics between the derivation and validation cohorts, we used the Student’s t-test for normally distributed continuous variables and the Mann–Whitney U test for non-normally distributed continuous variables. Differences in categorical variables were assessed using the Chi-square test. Statistical significance was determined with a two-sided *p* value less than 0.05.

The performance of risk of death prediction models was compared using classification accuracy, recall, precision, F1 score, and AUC-ROC. To test whether the differences between the results of risk of death prediction models were statistically significant, we used the Mann-Whitney U test  [48].

## Results

### Patient characteristics

A total of 16,018 patients requiring intensive care treatment and MV for at least 24 hours were enrolled in this study. The cohort was divided into two groups: 12,758 patients in the derivation cohort and 3,260 patients in the validation cohort. A comparison of clinical characteristics and outcomes between the two cohorts is presented in Tables 2 and 3, respectively.

### Structural knowledge representation

To represent structural knowledge in clinical feature spaces for in-ICU risk of death prediction, we applied fuzzy C-means clustering to preprocessed, vectorized textual data. These data, generated by GPT-4o, include medical information on the impact of each feature from Table 2 on ICU death rates. The clustering process was designed to identify distinct feature clusters based on the co-occurrence of mortality-associated keywords, representing different mortality-related patterns.

**Table 3:** Clinical outcomes

	Derivation (N=12758)	Validation (N=3260)	*P* value
Outcomes			
7-day death rate^a^, ^c^	1666 (13.1)	449 (13.8)	0.28
In-ICU death rate^a^	3645 (28.6)	1051 (32.2)	<0.05
ICU length of stay (days)^b^	10 (5-23)	12.48 (6.39-26.4)	<0.05

^a^ Presented as frequencies (percentages) and analyzed using the Chi-square test

^b^ Presented as median (interquartile range) and analyzed using the Mann–Whitney U test

^c^ from the start of mechanical ventilation

**Fig. 3:**
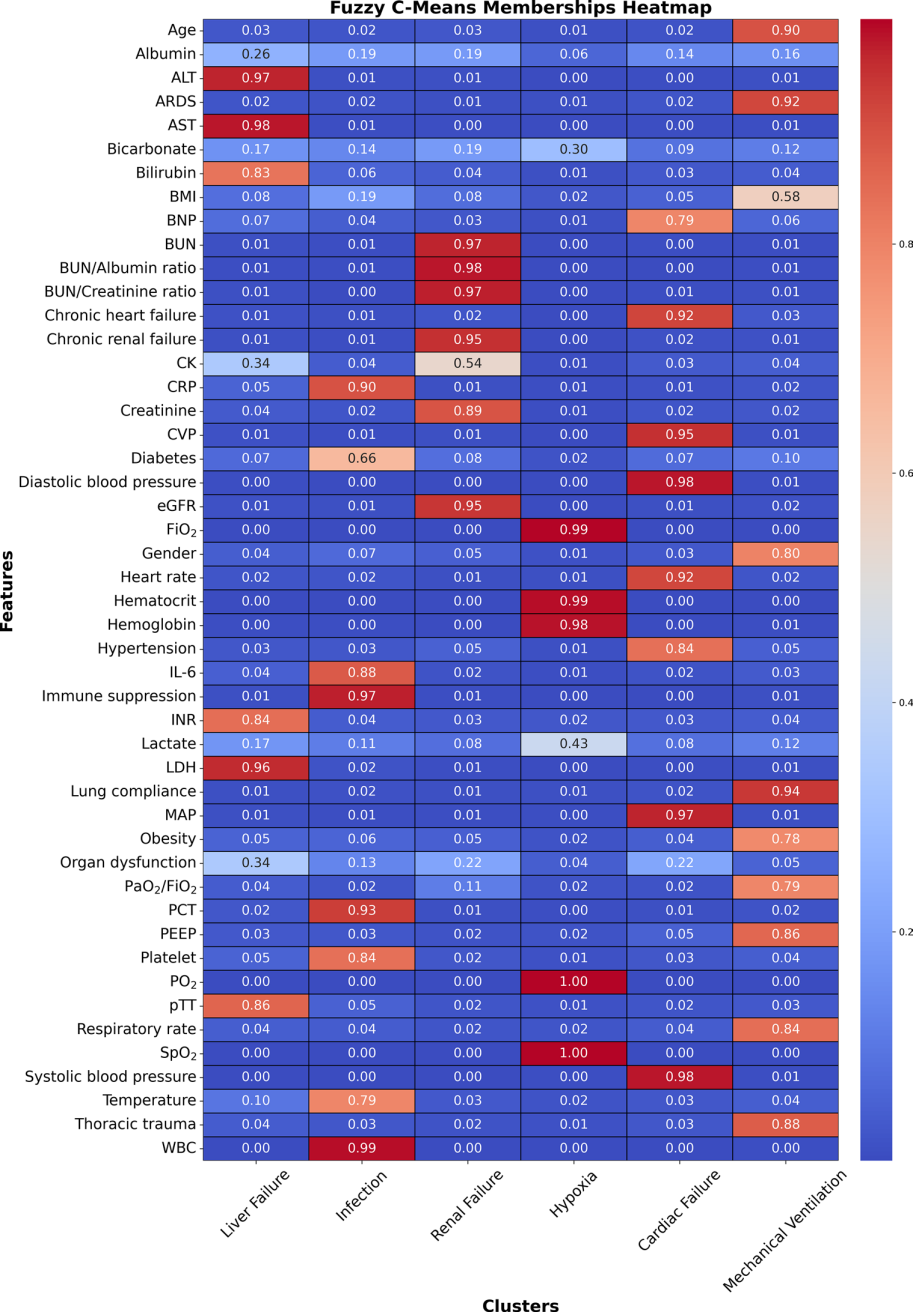
Heatmap illustrating fuzzy memberships of features within six clusters identified by fuzzy C-means clustering. Features with membership values above 0.2 are considered part of a cluster

The heatmap in Fig. 3 illustrates the fuzzy memberships of various features within six clusters. As fuzzy C-means is a soft clustering approach, it allows a feature to be included in more than one cluster. We determined the features included in each cluster by setting a cut-off value of 0.2 for the fuzzy memberships.

A physician then reviewed the features grouped in each cluster, confirming their relevance to specific mortality causes in mechanically ventilated ICU patients, and named the clusters accordingly. As a result, each cluster corresponded to a particular in-ICU mortality cause: liver failure, infection, renal failure, hypoxia, cardiac failure, and mechanical ventilation. Table 4 summarizes the resulting clusters and their associated features.

**Table 4:** Overview of the resulting clusters and their features

Cluster	Features
Liver Failure	Albumin, ALT, AST, Bilirubin, CK, INR, LDH, Organ dysfunction, pTT.
Infection	CRP, Diabetes, IL-6, Immune suppression, PCT, Platelet, Temperature, WBC.
Renal Failure	BUN, BUN/Albumin ratio, BUN/Creatinine ratio, Chronic renal failure, CK, Creatinine, eGFR, Organ dysfunction.
Hypoxia	Bicarbonate, FiO_2_, Hematocrit, Hemoglobin, Lactate, PO_2_, SpO_2_.
Cardiac Failure	BNP, Chronic heart failure, CVP, Diastolic blood pressure, Heart rate, Hypertension, MAP, Organ dysfunction, Systolic blood pressure.
Mechanical Ventilation	Age, ARDS, BMI, Gender, Lung compliance, Obesity, PaO_2_/FiO_2_, PEEP, Respiratory rate, Thoracic trauma.

### Predictive performances

We used the observational window data from the derivation cohort to train our GPT hybrid model, which includes six weak classifiers that were trained using distinct feature clusters in Table 4, and a strong classifier trained on the outputs of the weak classifiers. We benchmarked our model against the Global XGBoost classifier, which was trained on the complete feature set outlined in Table 2 without any pre-structuring. Both models underwent 5-fold stratified cross-validation with the derivation cohort’s data for hyperparameter tuning to ensure the best configuration.

**Fig. 4:**
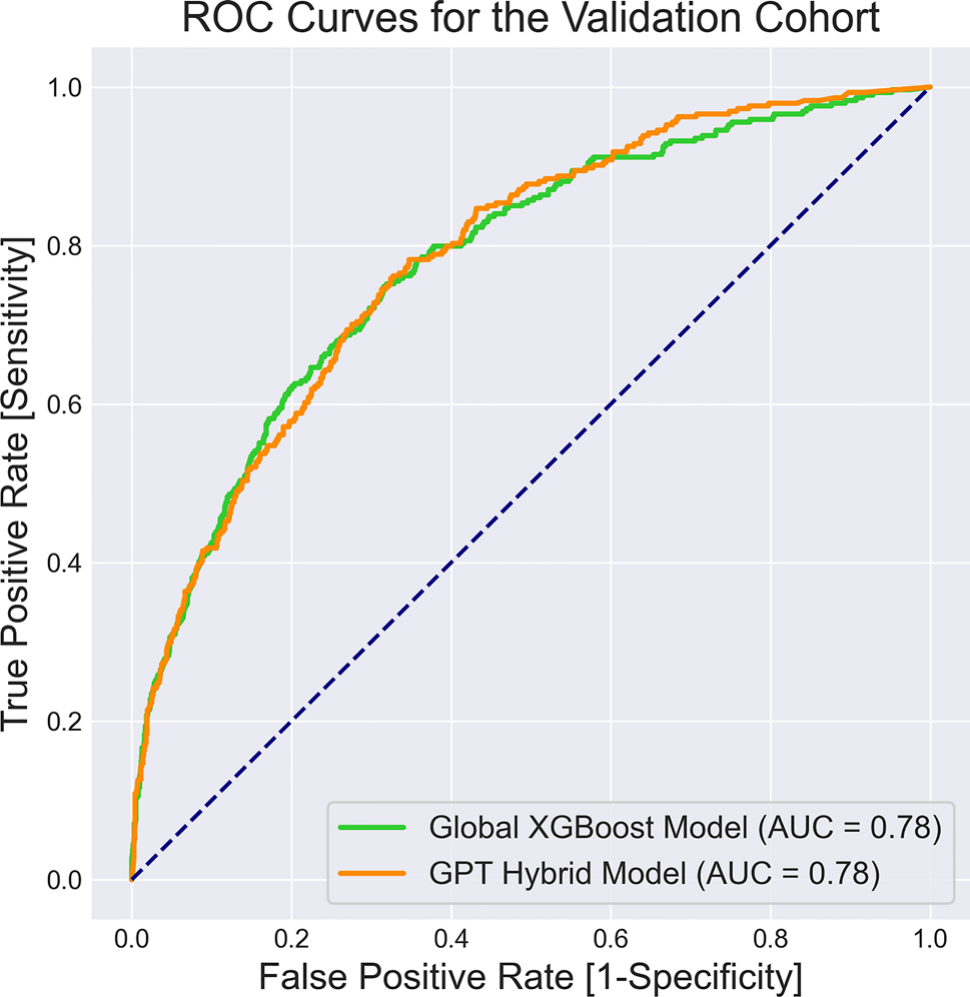
AUC-ROC curves comparing the performance of the GPT hybrid model and the global XGBoost model in predicting 7-day survival of ICU patients

The performance metrics for both predictive models, distinguishing between patients who survived and those who did not within six days of ICU stay following the observation window, are detailed in Table 5. Recall and precision metrics were calculated using prediction thresholds determined by maximizing accuracy on the derivation cohort (0.42 for the Global XGBoost model and 0.53 for the GPT hybrid model). These thresholds were then applied to the validation cohort. Additionally, Fig. 4 shows the AUC-ROC curves comparing the performance of the models. A Mann-Whitney U test with a significance level of 0.05 on these classification metrics revealed no significant difference in predictive accuracy between the GPT hybrid model and the Global XGBoost model during both the training and testing phases.

**Table 5:** Classification metrics for the developed GPT hybrid model and the Global XGBoost model for ICU risk of death prediction across derivation and validation cohorts

Data source	Classifier	Accuracy	Recall	Precision	ROC AUC
Derivation Cohort	Global XGBoost	0.899	0.620	0.750	0.885
	GPT Hybrid	0.905	0.653	0.819	0.918
Validation Cohort	Global XGBoost	0.879	0.270	0.622	0.780
	GPT Hybrid	0.873	0.424	0.541	0.784

### Feature importance analysis

**Fig. 5:**
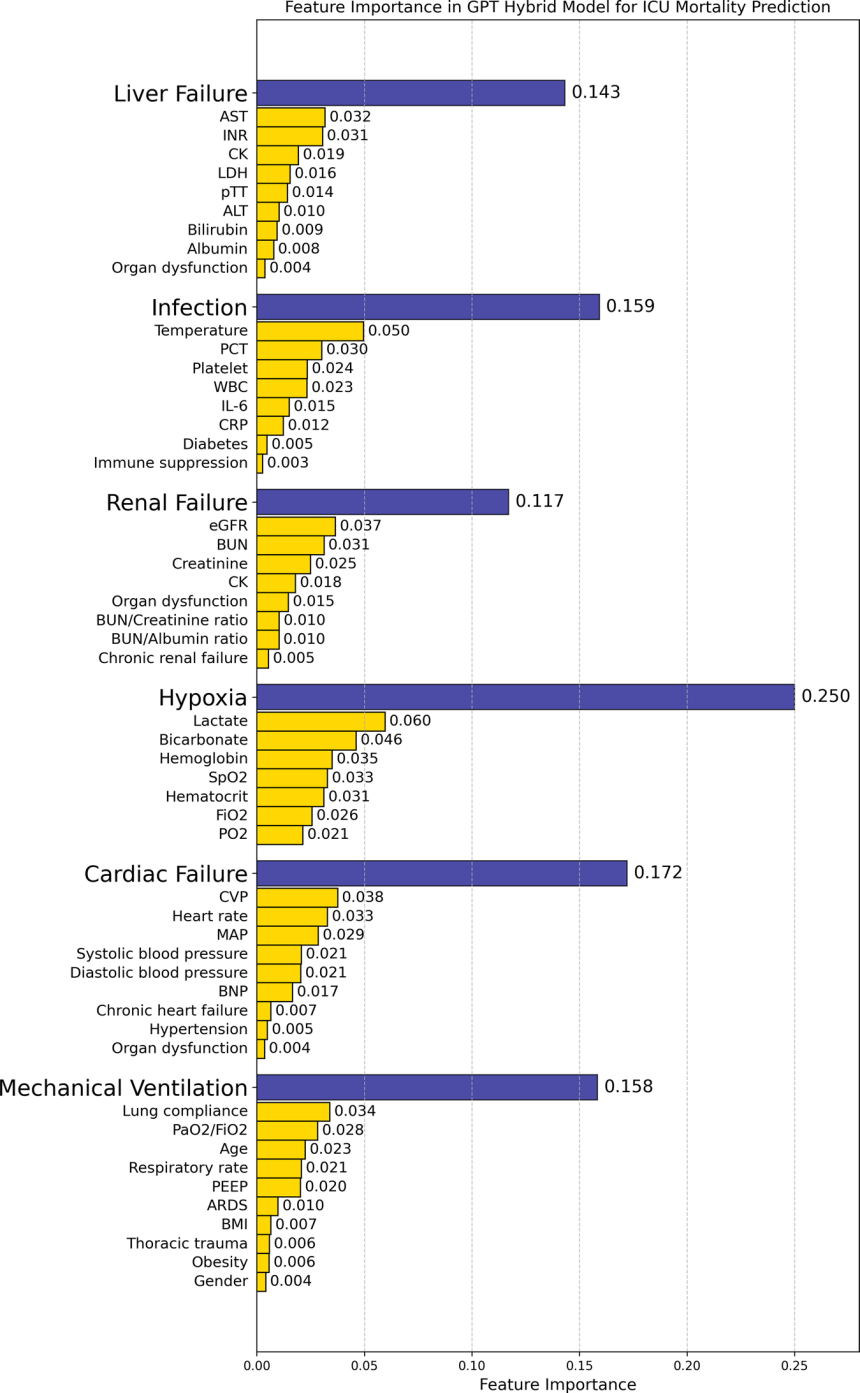
Feature importance in GPT hybrid model for ICU risk of death prediction. The chart displays a hierarchical structure of feature importance, with major mortality causes (blue bars) and their associated clinical parameters (yellow bars). This model incorporates a wide range of clinically relevant features across different causes of in-ICU mortality

**Fig. 6:**
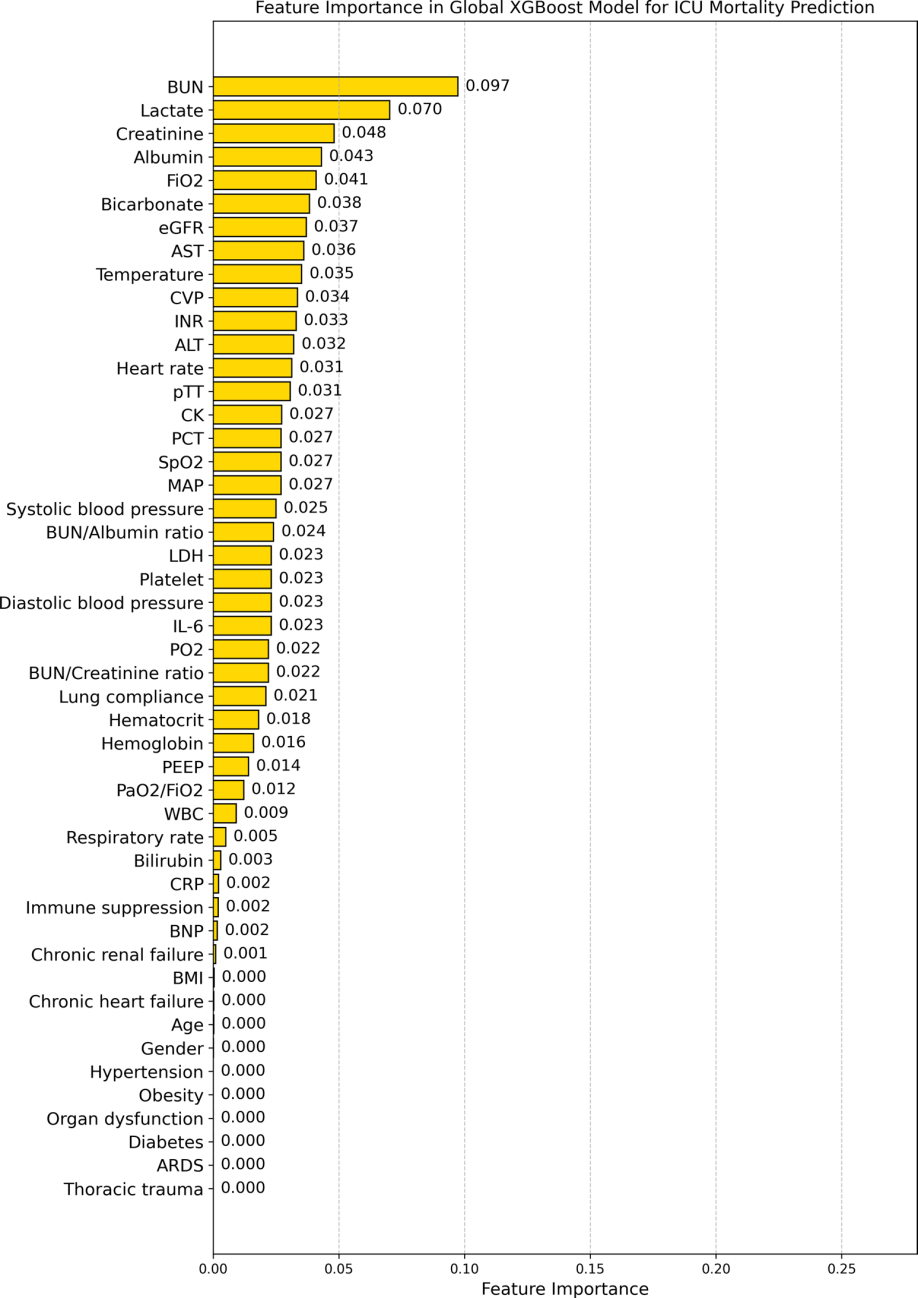
Feature importance in the global XGBoost for ICU risk of death prediction. The chart shows the relative importance of individual clinical parameters in the global XGBoost model’s risk of death predictions. Note the concentration of importance in a subset of features, with several clinically relevant factors showing minimal or zero importance

We compared the feature importance distributions between our GPT hybrid model and the Global XGBoost model for ICU risk of death prediction to assess their interpretability and clinical relevance (Figs. 5 and 6).

Our GPT hybrid model demonstrates a hierarchical structure of feature importance, see Fig. 5. At the top level, the model identifies the significance of each feature cluster listed in Table 4 for the final risk of death prediction executed by the strong classifier, with Hypoxia (0.250), Cardiac Failure (0.172), Infection (0.159), Mechanical Ventilation (0.158), Liver Failure (0.143), and Renal Failure (0.117). Within each feature cluster, individual clinical features show varied levels of importance as determined by the associated weak classifiers. For instance, under the Hypoxia cluster, Lactate (0.060) and Bicarbonate (0.046) are the most crucial indicators, while for the Cardiac Failure cluster, CVP (0.038) and Heart rate (0.033) play prominent roles.

This structured approach ensures that clinically relevant features of different mortality causes contribute to the predictions. Notably, the feature importance analysis of our model shows that it incorporates almost all the features listed in Table 2 in the risk of death risk prediction decision-making process.

In contrast, the Global XGBoost model exhibits a noticeably different feature importance distribution, as illustrated in Fig. 6. The top five most important features are BUN (0.097), Lactate (0.070), Creatinine (0.048), Albumin (0.043), and FiO2 (0.041). While these features are indeed clinically relevant, the Global XGBoost model assigns zero importance to several critical factors such as age, BMI, gender, ARDS, chronic heart failure, diabetes, hypertension, and organ dysfunction.

### Interpretability analysis

We demonstrated how GPT-4o can be used within our framework to create a hierarchically structured, cause-specific risk of death classifier. As a toy example to illustrate the resulting interpretability improvements, we performed a Shapley value analysis (using the SHAP library  [49]) on a representative patient from the validation cohort for whom both the Global XGBoost model and our GPT hybrid model yielded high-risk (and ultimately correct) risk of death predictions.

**Fig. 7:**
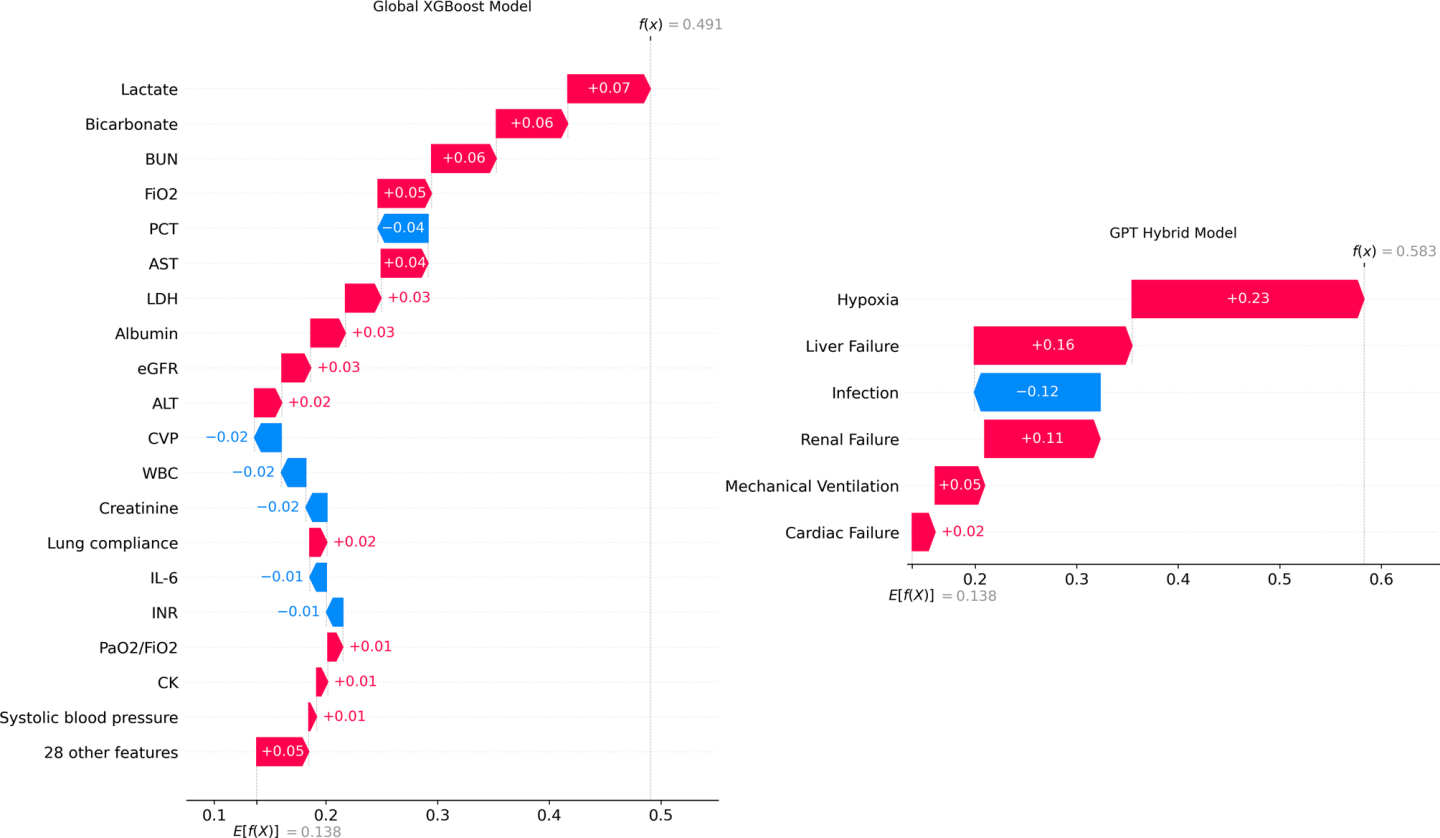
Waterfall plots of the SHAP-based explanations for a representative patient’s risk of death predictions. E[f(x)] denotes the model’s baseline output (the mortality average of the validation cohort), and f(x) is the model’s raw output (the risk of death for the patient). **Left**. global XGBoost model’s explanation, which assigns SHAP values to numerous individual features. **Right**. GPT hybrid model’s explanation, where feature clusters highlight cause-specific risk contributions. By focusing on a smaller set of feature clusters, the GPT hybrid model offers a more interpretable mapping from specific mortality factors to overall risk predictions

Figure 7 (left) shows the SHAP explanations for the Global XGBoost model. These explanations highlight numerous factors influencing the model’s outcomes, including a significant combined impact from 28 additional features not listed among the 20 most influential. The complex pattern of how various features affect the model’s output requires a medical expert to understand precisely how individual mortality factors drive the model’s prediction. Such complexity in the post-hoc explanations can undermine the purpose of AI-based clinical decision support systems, which is to give physicians and medical practitioners clear, actionable insights.

In contrast, the GPT hybrid model’s outcome explanations focus on interactions among feature clusters rather than considering all features at once. As shown in Fig. 7 (right), the SHAP values clarify each feature cluster’s contribution to the model outcome, specifically illustrating how individual mortality factors drive the model’s predictions. This localized perspective mitigates the uncertainty that arises from global feature interactions, enabling a direct mapping from specific mortality causes in mechanically ventilated ICU patients to the model’s risk predictions. Consequently, the model’s outputs become easier for physicians and medical practitioners to interpret, offering actionable insights for clinical decision support.

To evaluate the clinical validity of the automatically derived mortality cause-specific clusters and their contributions to ICU mortality, we confirmed that, given unlimited time, an experienced intensive care physician would likely organize a similar set of variables into the six pathophysiological categories identified in this study. The principal advantage of the proposed framework is time: the cluster-based display delivers equivalent insights in a fraction of the time required for manual synthesis. Additionally, the cluster-level representation is particularly beneficial for less-experienced clinicians, as it presents related laboratory and monitoring data in an integrated, disease-oriented format, thus reducing the cognitive burden of interpreting numerous isolated measurements.

## Discussion

In this study, we utilized GPT-4o to generate detailed medical feature descriptions for structural knowledge representation, enabling the identification of significant mortality cause-specific feature clusters. These clusters informed the creation of a structured and therefore interpretable GPT hybrid model for ICU risk of death prediction. Our results show that the performance of our model in ICU risk of death prediction for mechanically ventilated patients is comparable to the Global XGBoost model, demonstrating that an interpretable modeling workflow does not compromise predictive accuracy. Additionally, our model’s integration of domain knowledge ensures comprehensive ICU risk of death prediction by including a wide array of critical features, which is not the case in the Global XGBoost model.

The primary advantage of using GPT-4o in this study is the implementation of an automated process for clustering mortality cause-specific features, thereby reconstructing a hierarchically structured hybrid model for ICU risk of death prediction with enhanced interpretability. Previous structured hybrid modeling approaches for ICU risk of death prediction in COVID-19  [17] and mechanically ventilated influenza  [18] patients relied on manual trial-and-error and selective feature identification. In contrast, our work offers a fully automated process for reconstructing a hierarchically structured hybrid model. Although this automation saves time and effort, it still requires meaningful clinical oversight to ensure that automatically identified features are both relevant and actionable.

Moreover, while expert-defined clinical groupings and traditional clustering algorithms have their merits, we chose GPT–based clustering as a practical and efficient alternative. Relying on expert consensus is time-intensive and often leads to multiple, inconsistent classifications due to varying clinical practices. Traditional algorithms like k-means focus on statistical co-variation in patient data, which may not capture the clinical meaning essential for interpretability. In contrast, our method clusters features based on semantic similarity from LLM-generated descriptions, emphasizing clinical relevance.

Here, we would like to highlight three aspects of our contribution:

First, we present a comprehensive workflow for utilizing LLMs to develop an interpretable modeling framework aimed at predicting ICU risk of death among mechanically ventilated patients. By systematically leveraging the capabilities of LLMs, the study demonstrates how complex clinical features can be represented and organized into meaningful mortality-cause-specific clusters. We then employ a novel risk of death classification technique that leverages boosting on the resulting feature clusters to enhance interpretability. This approach not only provides a deeper understanding of the underlying predictors of risk of death but also sets the stage for applying interpretable artificial intelligence models in other medical domains.

Second, our cluster analysis depicted the structural medical knowledge within the clinical feature space of this study by identifying six distinct feature groups (see Table 4). Each of these groups reflects key causes of mortality in mechanically ventilated ICU patients:

- Cluster 1 features capture complications associated with liver failure. AST and ALT levels indicate liver cell damage, while Bilirubin levels reflect the liver’s impaired excretion functions. Additionally, PTT and INR values reveal the liver’s failure to produce coagulation factors. The organ dysfunction flag is crucial, as the liver is often one of the last organs to fail before death.
- Cluster 2 features are linked to infection parameters. WBC, PCT, IL-6, CRP, and Temperature are direct indicators of infection. Additionally, diabetes and immune suppression highlight susceptibility to infection. Furthermore, platelet levels often drop in sepsis, indicating the progression and severity of the infectious process.
- Cluster 3 includes features associated with renal failure. eGFR, creatinine, BUN, BUN/albumin ratio, and BUN/creatinine ratio reflect impaired kidney excretion functions. Chronic renal failure indicates long-term kidney impairment, while a significant elevation in CK serves as a risk factor for acute renal failure.
- Cluster 4 encompasses hypoxia, characterized by impaired pulmonary oxygenation indicated by parameters such as PO_2_, FiO_2_, and SpO_2_, as well as impaired oxygen transfer into the tissue evidenced by hemoglobin and hematocrit levels. Additionally, hypoxia involves elevated lactate levels, which are a marker of anaerobic metabolism, and bicarbonate levels, which are elevated in long-term respiratory failure as compensation for respiratory acidosis.
- Cluster 5 features reflect cardiac failure, as indicated by altered values in systolic blood pressure, MAP, diastolic arterial pressure, CVP, and heart rate, which are associated with failing pump function. Additionally, the lab value of BNP is indicative of heart failure. Hypertension is noted as one of the strongest risk factors for cardiac failure, and chronic heart failure is also present within this cluster.
- Cluster 6 includes features associated with mechanical ventilation, such as PaO2/FiO2, respiratory rate, and PEEP, which are typical parameters relevant to this treatment. Furthermore, risk factors for requiring mechanical ventilation include thoracic trauma, obesity, high BMI, ARDS, age, and gender. Additionally, lung compliance, which varies in pulmonary diseases, is an important aspect to consider in this context.

While the clinical validity of our cluster-based explanations is encouraging, we have not yet conducted a formal user study; future work is therefore needed to quantify clinician comprehension and downstream decision impact.

Third, the feature importance analysis demonstrated that the GPT hybrid model is more reliable and clinically relevant compared to the Global XGBoost approach for predicting ICU risk of death. The feature importance in the Global XGBoost model appears to be primarily driven by statistical patterns in the given training dataset, potentially at the expense of clinical comprehensiveness. In contrast, our model balances statistical learning with provided structural medical knowledge, potentially offering greater reliability by considering a wide range of clinical features and their related causes of mortality in the risk of death predictions. This balance does not compromise predictive accuracy, as confirmed by the predictive performance analysis.

This study’s limitations present opportunities for future research and improvements in the application of LLMs to clinical prediction tasks. A primary concern is the phenomenon of “hallucination” in LLMs, where the model generates outputs that sound plausible but are factually incorrect or unsupported by its training data. This issue arises particularly when the model is prompted to provide information beyond the scope of its knowledge base. Although our workflow does not directly use GPT-generated information for clinical decision-making, it does rely on these outputs to identify structural patterns within the feature space for our boosting technique. A further and equally important constraint is that our model development and validation were performed using data drawn from a single hospital. As a result, the external validity and portability of the proposed workflow to other hospitals, geographic regions, and patient populations remain untested. Future studies should therefore incorporate multicenter datasets or prospective external validation cohorts before any clinical deployment is considered.

Hallucinations in this study were largely due to the model producing incorrect, non-relevant, or misleading information, so we implemented a multi-step approach to mitigate them. First, we performed a validity check by reviewing the texts and removing potentially incorrect information, such as suggesting a direct relationship between FiO_2_ levels and acute ARDS due to oxygen toxicity, which is a misrepresentation since ARDS is a complex condition with multiple causes. Second, we applied TF-IDF vectorization to the preprocessed textual data and then performed distance-based clustering using Fuzzy C-means. As explained in the Methods section, TF-IDF measures each word’s importance in describing a feature and how unique it is across the corpus; hence, TF-IDF scores for non-relevant information (for example, overstating the significance of CK levels in indicating myocardial infarction or rhabdomyolysis) remain low, reducing their distance impact in clustering. Third, we finalized feature clustering by analyzing fuzzy memberships. Certain misleading hallucinations (for example, describing the PaO_2_/FiO_2_ ratio as though it were commonly combined with physiological parameters such as the BUN/Albumin ratio) could lead to small fuzzy memberships for otherwise unrelated clusters, so we introduced a minimum membership threshold to help ensure features are placed in relevant clusters. Finally, we used expert analysis of the resulting clusters to interpret their associated causes of mortality. Nevertheless, residual LLM hallucinations or hidden knowledge biases may persist despite our manual review and clustering safeguards; complementary automated fact-checking algorithms should be explored in future iterations to further mitigate this risk.

Our proposed modeling framework also presents challenges when incorporating new features. Unlike traditional machine learning approaches, adding new features to our model requires implementing all steps prior to training the boosting model. This ensures the proper inclusion of new features in existing clusters or the reshaping of the feature clusters themselves. This limitation highlights the importance of comprehensive initial feature selection and the need for efficient methods to update the model as new relevant features are identified. Furthermore, as the model performance was assessed on a single, time-held-out validation cohort, we did not calculate confidence intervals; standard resampling techniques would violate the temporal independence of this split and risk optimistic bias. Future work using larger, multi-center datasets will permit repeated sampling and formal uncertainty quantification.

We utilized fuzzy C-means primarily because it allows each feature partial membership in multiple clusters. This flexibility is crucial to our investigation of clustering clinical features by their impact on ICU risk of death, since forcing a feature into just one cluster can be overly restrictive. For instance, the organ dysfunction flag could belong to both a liver failure cluster and a renal failure cluster. Moreover, by examining the membership values produced by fuzzy C-means, one can determine whether a feature aligns closely with a single cluster or shows comparable similarity to multiple clusters. This approach helps clinicians better understand the extracted multifactorial influences of these features on ICU risk of death. Despite these benefits, fuzzy C-means has some limitations. The fuzziness parameter influences how much overlap is allowed and needs to be selected carefully to fit the complexity of the data. Additionally, the algorithm can be more computationally intensive than traditional hard clustering methods.

There are also challenges associated with integrating novel AI-based predictive models into ICU settings. Although ICUs are typically considered a technophile environment, the adoption of AI-based systems remains limited, mainly due to insufficient evidence that these models indeed improve patient outcomes. Generating such evidence is therefore critical before implementing AI in ICU therapy. This process should begin with extensive in silico testing using virtual patient data that represents a broad range of conditions, starting with low-risk patients and gradually moving toward more complex, critically ill ICU populations. Subsequently, prospective clinical trials—ideally randomized against existing standard-of-care protocols—should be conducted, followed by transparent and thorough discussions of the results.

It is important to recognize that implementing AI models in the ICU requires physicians to hand over a significant part of their decision-making process. They will only accept this transition if they are confident that it will not compromise patient safety. Enhancing the interpretability of AI models can greatly help physicians embrace this “sharing of responsibility,” though the ultimate responsibility must always remain with the physician. Moreover, AI can be extremely valuable for teaching and training early-career physicians or medical students. An interpretable AI model can highlight early signs of deterioration in a specific organ system - signals that might otherwise go unnoticed. This, in turn, helps medical staff focus on emerging issues and take appropriate measures in a timely manner. However, our observations qualitatively support the interpretability of the proposed approach and have limitations. Formal user studies will be necessary in the future to quantify the claimed time savings and their impact on clinical decision-making.

In line with recent work  [50, 51], integrating risk-based predictions with continuous bedside monitoring may enable dynamic adjustments of alarm thresholds and volumes based on patient-specific, time-varying risks. Such an approach can help reduce alarm fatigue while enabling more targeted and timely support for high-risk patients, ultimately improving outcomes and guiding individualized care in critical care settings. Nevertheless, because our study leveraged large, heterogeneous datasets without full information on therapy decisions, it does not provide the granular detail required for patient-level interventions, such as earlier palliative care involvement or other ICU-specific measures aimed at reducing mortality in practice.

Lastly, our approach primarily focuses on mechanically ventilated patients, potentially limiting its transferability to other ICU patient groups. Adapting the model to diverse patient populations will require modified prompting strategies and expert evaluation of the resulting feature clusters. This opens up new avenues for future work in developing a generalizable workflow for extracting medical knowledge across diverse ICU patient groups.

## Conclusion

This study presents a novel approach to predicting ICU risk of death for mechanically ventilated patients using a GPT hybrid model. By integrating LLMs with traditional machine learning techniques, our methodology demonstrates the potential to create interpretable and clinically relevant predictive models. The introduced GPT hybrid model achieved comparable performance to a Global XGBoost model, indicating that increased interpretability does not come at the cost of predictive accuracy. More importantly, our model’s feature importance analysis revealed a more comprehensive and clinically coherent utilization of relevant factors compared to the Global XGBoost model. Future research should focus on developing more flexible feature incorporation methods and expanding the model’s applicability to diverse patient populations.

## Electronic supplementary material

Below is the link to the electronic supplementary material.

Supplementary Material 1

## Data Availability

The data included in this study contain sensitive health-related information. Due to the small data set, anonymisation techniques, like e.g. k-anonymity, cannot be applied usefully without a relevant loss of information. Thus, according to the Health Data Protection Act North Rhine-Westphalia (Gesundheitsdatenschutzgesetz NRW) and the internal guidelines of the Data Protection Officer of the University Hospital RWTH Aachen, the raw patient data must not be made publicly available, since a total anonymisation cannot be guaranteed. However, researchers who are interested in the data may send their informal request to the Department of Intensive Care Medicine (Email: oim@ukaachen.de) of the University Hospital RWTH Aachen with a statement which research questions they aim at and which data are necessary for this purpose. Then, in a bilateral process, a solution for the data exchange can be found in compliance with legal and ethical restrictions.

## Abbreviations

ASIC: Algorithmic Surveillance of ICU Patients with Acute Respiratory Distress Syndrome
ALT: Alanine Aminotransferase
ARDS: Acute Respiratory Distress Syndrome
AST: Aspartate transaminase
AUC-ROC: Area under the receiver operating characteristic curve
BNP: B-type Natriuretic Peptide
BUN: Blood Urea Nitrogen
CK: Creatine Kinase
CRP: C-Reactive Protein
CVP: Central Venous Pressure
eGFR: estimated Glomerular Filtration Rate
FiO_2_: Fraction of Inspired Oxygen
ICU: Intensive care unit
IL-6: Interleukin 6
INR: International Normalized Ratio
LDH: Lactate Dehydrogenase
MAP: Mean Arterial Pressure
MV: Mechanical Ventilation
PaO_2_/FiO_2_: Partial Pressure of Arterial Oxygen to Fraction of Inspired Oxygen Ratio
PCT: Procalcitonin
PEEP: Positive End-Expiratory Pressure
PO_2_: Partial Pressure of Oxygen
pTT: Partial Thromboplastin Time
SpO_2_: Peripheral Oxygen Saturation
TF-IDF: Term Frequency-Inverse Document Frequency
WBC: White Blood Cell Count
XGBoost: Extreme gradient boosting
