# Supplementary material for: GPT-4o and the quest for machine learning interpretability in ICU risk of death prediction
Source: BMC Med Inform Decis Mak. 2025 Oct 13;25:373. doi: 10.1186/s12911-025-03224-z (PMC12516888; doi:10.1186/s12911-025-03224-z)
Supplement: Supplementary file 1 — Supplementary Material 1 [file 12911_2025_3224_MOESM1_ESM.pdf]

Section 1 — TRIPOD+AI checklist

Section 1 of the Supplementary Material presents the completed TRIPOD-AI checklist for this study, with item-by-item responses, applicability notes (N/A where appropriate), and corresponding page/section citations to the main manuscript.

TRIPOD+AI

Version: 11-January-2024

Section/Topic	Item	Development / evaluation[1]	Checklist item	Reported on page
**TITLE**				
Title	1	D;E	Identify the study as developing or evaluating the performance of a multivariable prediction model, the target population, and the outcome to be predicted	1
**ABSTRACT**				
Abstract	2	D;E	See TRIPOD+AI for Abstracts checklist	1
**INTRODUCTION**				
Background	3a	D;E	Explain the healthcare context (including whether diagnostic or prognostic) and rationale for developing or evaluating the prediction model, including references to existing models	2
	3b	D;E	Describe the target population and the intended purpose of the prediction model in the context of the care pathway, including its intended users (e.g., healthcare professionals, patients, public)	2
	3c	D;E	Describe any known health inequalities between sociodemographic groups	2
Objectives	4	D;E	Specify the study objectives, including whether the study describes the development or validation of a prediction model (or both)	3, 5
**METHODS**				
Data	5a	D;E	Describe the sources of data separately for the development and evaluation datasets (e.g., randomised trial, cohort, routine care or registry data), the rationale for using these data, and representativeness of the data	3
	5b	D;E	Specify the dates of the collected participant data, including start and end of participant accrual; and, if applicable, end of follow-up	3
Participants	6a	D;E	Specify key elements of the study setting (e.g., primary care, secondary care, general population) including the number and location of centres	3
	6b	D;E	Describe the eligibility criteria for study participants	3
	6c	D;E	Give details of any treatments received, and how they were handled during model development or evaluation, if relevant	N/A
Data preparation	7	D;E	Describe any data pre-processing and quality checking, including whether this was similar across relevant sociodemographic groups	3, 4
Outcome	8a	D;E	Clearly define the outcome that is being predicted and the time horizon, including how and when assessed, the rationale for choosing this outcome, and whether the method of outcome assessment is consistent across sociodemographic groups	5
	8b	D;E	If outcome assessment requires subjective interpretation, describe the qualifications and demographic characteristics of the outcome assessors	N/A
	8c	D;E	Report any actions to blind assessment of the outcome to be predicted	N/A
Predictors	9a	D	Describe the choice of initial predictors (e.g., literature, previous models, all available predictors) and any pre-selection of predictors before model building	4, 5
	9b	D;E	Clearly define all predictors, including how and when they were measured (and any actions to blind assessment of predictors for the outcome and other predictors)	4, 5
	9c	D;E	If predictor measurement requires subjective interpretation, describe the qualifications and demographic characteristics of the predictor assessors	N/A
Sample size	10	D;E	Explain how the study size was arrived at (separately for development and evaluation), and justify that the study size was sufficient to answer the research question. Include details of any sample size calculation	3, 4
Missing data	11	D;E	Describe how missing data were handled. Provide reasons for omitting any data	3
Analytical methods	12a	D	Describe how the data were used (e.g., for development and evaluation of model performance) in the analysis, including whether the data were partitioned, considering any sample size requirements	3
	12b	D	Depending on the type of model, describe how predictors were handled in the analyses (functional form, rescaling, transformation, or any standardisation).	5-7
	12c	D	Specify the type of model, rationale[2], all model-building steps, including any hyperparameter tuning, and method for internal validation	5-7
	12d	D;E	Describe if and how any heterogeneity in estimates of model parameter values and model performance was handled and quantified across clusters (e.g., hospitals, countries). See TRIPOD-Cluster for additional considerations[3]	N/A
	12e	D;E	Specify all measures and plots used (and their rationale) to evaluate model performance (e.g., discrimination, calibration, clinical utility) and, if relevant, to compare multiple models	11
	12f	E	Describe any model updating (e.g., recalibration) arising from the model evaluation, either overall or for particular sociodemographic groups or settings	N/A
	12g	E	For model evaluation, describe how the model predictions were calculated (e.g., formula, code, object, application programming interface)	N/A
Class imbalance	13	D;E	If class imbalance methods were used, state why and how this was done, and any subsequent methods to recalibrate the model or the model predictions	N/A
Fairness	14	D;E	Describe any approaches that were used to address model fairness and their rationale	14, 16
Model output	15	D	Specify the output of the prediction model (e.g., probabilities, classification). Provide details and rationale for any classification and how the thresholds were identified	9, 16, 17

Training versus evaluation	16	D;E	Identify any differences between the development and evaluation data in healthcare setting, eligibility criteria, outcome, and predictors	N/A
Ethical approval	17	D;E	Name the institutional research board or ethics committee that approved the study and describe the participant-informed consent or the ethics committee waiver of informed consent	17, 18
**OPEN SCIENCE**				
Funding	18a	D;E	Give the source of funding and the role of the funders for the present study	18
Conflicts of interest	18b	D;E	Declare any conflicts of interest and financial disclosures for all authors	18
Protocol	18c	D;E	Indicate where the study protocol can be accessed or state that a protocol was not prepared	N/A
Registration	18d	D;E	Provide registration information for the study, including register name and registration number, or state that the study was not registered	N/A
Data sharing	18e	D;E	Provide details of the availability of the study data	18
Code sharing	18f	D;E	Provide details of the availability of the analytical code[4]	18
**PATIENT & PUBLIC INVOLVEMENT**				
Patient & Public Involvement	19	D;E	Provide details of any patient and public involvement during the design, conduct, reporting, interpretation, or dissemination of the study or state no involvement.	N/A
**RESULTS**				
Participants	20a	D;E	Describe the flow of participants through the study, including the number of participants with and without the outcome and, if applicable, a summary of the follow-up time. A diagram may be helpful.	3, 4, 8, 9
	20b	D;E	Report the characteristics overall and, where applicable, for each data source or setting, including the key dates, key predictors (including demographics), treatments received, sample size, number of outcome events, follow-up time, and amount of missing data. A table may be helpful. Report any differences across key demographic groups.	8, 9
	20c	E	For model evaluation, show a comparison with the development data of the distribution of important predictors (demographics, predictors, and outcome).	11-13
Model development	21	D;E	Specify the number of participants and outcome events in each analysis (e.g., for model development, hyperparameter tuning, model evaluation)	8, 9
Model specification	22	D	Provide details of the full prediction model (e.g., formula, code, object, application programming interface) to allow predictions in new individuals and to enable third-party evaluation and implementation, including any restrictions to access or re-use (e.g., freely available, proprietary)[5]	5-7
Model performance	23a	D;E	Report model performance estimates with confidence intervals, including for any key subgroups (e.g., sociodemographic). Consider plots to aid presentation.	9-13
	23b	D;E	If examined, report results of any heterogeneity in model performance across clusters. See TRIPOD Cluster for additional details[3].	N/A
Model updating	24	E	Report the results from any model updating, including the updated model and subsequent performance	N/A
**DISCUSSION**				
Interpretation	25	D;E	Give an overall interpretation of the main results, including issues of fairness in the context of the objectives and previous studies	14-17
Limitations	26	D;E	Discuss any limitations of the study (such as a non-representative sample, sample size, overfitting, missing data) and their effects on any biases, statistical uncertainty, and generalizability	16, 17
Usability of the model in the context of current care	27a	D	Describe how poor quality or unavailable input data (e.g., predictor values) should be assessed and handled when implementing the prediction model	16, 17
	27b	D	Specify whether users will be required to interact in the handling of the input data or use of the model, and what level of expertise is required of users	N/A
	27c	D;E	Discuss any next steps for future research, with a specific view to applicability and generalizability of the model	17

Section 2 — Prompt design and the generated medical feature descriptions using GPT-4o

Section 2 of the Supplementary Material provides detailed information on the prompt design used in our large language model (LLM) assisted methodology for ICU mortality prediction among mechanically ventilated patients.

Our innovative approach leverages GPT-4o to create detailed descriptions of medical features. These descriptions offer insights into how each feature listed in Table 1 of the main manuscript affects ICU mortality rates. We then compile these AI-generated descriptions into a corpus database, which serves as the foundational dataset for structural knowledge representation.

The following sections will delve into the specific prompt design that enables this process, demonstrating how we systematically extract and structure relevant medical information.

Prompt Design

Our prompt design follows a structured format to ensure consistent and relevant responses from the LLM. The general structure of our prompts is as follows:

```
Answer this question in [word count] words: How does [Medical Feature] impact
the ICU mortality rates of mechanically ventilated patients?
```

Key elements of this prompt design include:

1. **Specific word count**: This ensures responses are detailed enough to capture important information while remaining concise.
2. **Focus on a single medical feature**: This allows for in-depth exploration of each feature's impact.
3. **Target population specification**: By explicitly mentioning "mechanically ventilated patients," we ensure the response is tailored to our specific study population.

This prompt design allows us to systematically gather detailed information on each medical feature's impact on ICU mortality rates for mechanically ventilated patients.

Example Prompt and Response

Here's an example of a prompt used in our methodology, along with the corresponding response from GPT-4o:

Prompt:

```
Answer this question in 200 words: How does Age impact the ICU mortality rates
of mechanically ventilated patients?
```

Response:

"Age is a significant factor influencing the mortality rates of mechanically ventilated patients in Intensive Care Units (ICUs). As patients age, their physiological reserves diminish, and their ability to recover from critical illness decreases. This vulnerability is particularly evident in the context of mechanical ventilation, which is often required for severe respiratory failure. Elderly patients, generally those aged 65 and older, tend to have higher ICU mortality rates when subjected to mechanical ventilation compared to younger patients. This increased risk is due in part to the higher prevalence of comorbidities such as cardiovascular disease, diabetes, and chronic respiratory conditions in older populations. These comorbidities complicate the management of acute conditions and reduce the overall chances of survival. Studies have shown that the mortality rate for mechanically ventilated elderly patients can be significantly higher, especially when ventilation is needed for extended periods. The stress of mechanical ventilation can lead to complications like ventilator-associated pneumonia, barotrauma, and muscle weakness, all of which contribute to an increased risk of death. Moreover, age-related changes in lung physiology, such as decreased lung elasticity and impaired gas exchange, make older adults more susceptible to ventilator-induced lung injury. As a result, the management of mechanical ventilation in elderly patients requires careful balancing of ventilatory support to minimize these risks while providing sufficient respiratory assistance. In summary, age significantly impacts ICU mortality rates for mechanically ventilated patients, with older adults facing higher risks due to diminished physiological capacity and greater prevalence of complicating comorbidities."

Here is the generated corpus database for all features listed in Table 1 of the main manuscript:

"**Age:** Age is a significant factor influencing the mortality rates of mechanically ventilated patients in Intensive Care Units (ICUs). As patients age, their physiological reserves diminish, and their ability to recover from critical illness decreases. This vulnerability is particularly evident in the context of mechanical ventilation, which is often required for severe respiratory failure. Elderly patients, generally those aged 65 and older, tend to have higher ICU mortality rates when subjected to mechanical ventilation compared to younger patients. This increased risk is due in part to the higher prevalence of comorbidities such as cardiovascular disease, diabetes, and chronic respiratory conditions in older populations. These comorbidities complicate the management of acute conditions and reduce the overall chances of survival. Studies have shown that the mortality rate for mechanically ventilated elderly patients can be significantly higher, especially when ventilation is needed for extended periods. The stress of mechanical ventilation can lead to complications like ventilator-associated pneumonia, barotrauma, and muscle weakness, all of which contribute to an increased risk of death. Moreover, age-related changes in lung physiology, such as decreased lung elasticity and impaired gas exchange, make older adults more susceptible to ventilator-induced lung injury. As a result, the management of mechanical ventilation in elderly patients requires careful balancing of ventilatory support to minimize these risks while providing sufficient respiratory assistance. In summary, age significantly impacts ICU mortality rates for mechanically ventilated patients, with older adults facing higher risks due to diminished physiological capacity and greater prevalence of complicating comorbidities.",

"**Gender:** Gender is an important determinant in the ICU mortality rates of mechanically ventilated patients, influenced by biological and possibly social factors. Research indicates that there are differences in outcomes for men and women, although the findings can be complex and sometimes contradictory. Biologically, men and women differ in lung anatomy, immune response, and hormonal environment, all of which can influence their response to critical illness and mechanical ventilation. Men typically have larger lung volumes and airway diameters, which could affect the development of ventilator-induced lung injuries and other complications. Additionally, hormonal differences, particularly the presence of estrogen in women, might offer some degree of protection against inflammation and sepsis, which are common in critical illnesses requiring mechanical ventilation. Epidemiological studies often report that men have higher ICU mortality rates than women when all other factors are controlled. However, the picture is nuanced. For instance, the impact of gender on mortality can vary with age and the presence of other health conditions. Women may experience better survival rates in some settings, but this advantage diminishes with age, especially after menopause, suggesting that hormonal factors play a role in these outcomes. Moreover, social factors such as differences in health-seeking behavior, access to care, and pre-hospital health status might also influence outcomes. Overall, while gender impacts ICU mortality rates, the relationship is complex and influenced by an interplay of biological, clinical, and social factors.",

"**BMI:** Body Mass Index (BMI) significantly influences ICU mortality rates in mechanically ventilated patients, manifesting through various biological and clinical pathways. BMI categories—underweight, normal weight, overweight, and obese—each carry distinct risks and implications for mechanically ventilated patients. Patients with a low BMI (underweight) typically face higher mortality rates in the ICU. This group may have less physiological reserve and could be more susceptible to the negative effects of severe illnesses that necessitate mechanical ventilation. Underweight patients often have poorer nutritional status, which can impair immune function and wound healing, increasing susceptibility to infections and other complications. Conversely, overweight and obese patients often show a paradoxically lower mortality rate in the ICU—a phenomenon sometimes referred to as the obesity paradox. These patients may benefit from greater metabolic reserves during critical illness, which could provide enhanced survival during acute episodes requiring mechanical ventilation. However, managing ventilation for obese patients is technically challenging due to issues like decreased lung compliance, increased airway resistance, and higher prevalence of obesity-related comorbidities such as cardiovascular disease and diabetes. Despite the protective effect seen in acute settings, the long-term outcomes for obese patients may still be unfavorable. The initial survival advantage could be offset by increased rates of complications such as infections and prolonged hospital stays. Overall, BMI plays a complex role in influencing ICU outcomes for mechanically ventilated patients, with varying impacts depending on whether a patient is underweight, normal weight, or obese.",

"**eGFR:** The estimated glomerular filtration rate (eGFR), a measure of kidney function, is a critical factor influencing ICU mortality rates in mechanically ventilated patients. eGFR indicates how well the kidneys are filtering blood and removing waste products. Lower eGFR values suggest impaired kidney function, which is linked to higher mortality rates in critical care settings. In ICU patients, particularly those requiring mechanical ventilation, reduced kidney function as indicated by a low eGFR can lead to several complications. These patients are more likely to experience fluid overload, electrolyte imbalances, and accumulation of toxic waste products, all of which can exacerbate the underlying illness and complicate the management of ventilation and other critical care interventions. Moreover, a decreased eGFR often correlates with the presence of other serious health conditions such as chronic kidney disease, diabetes, and hypertension, which can independently increase mortality risk. The kidneys play a vital role in drug metabolism and excretion; thus, impaired kidney function complicates the pharmacokinetic management of drugs used in critical care, including dosages of antibiotics, sedatives, and other medications essential for

the management of ventilated patients. Overall, a lower eGFR is associated with poorer outcomes and higher mortality rates in the ICU, highlighting the importance of renal function as a prognostic indicator in critically ill, mechanically ventilated patients. This relationship underscores the need for meticulous monitoring and management of renal function to improve survival chances in this vulnerable population.",
"**PO2:** Partial pressure of oxygen (PO2) levels in the blood is a crucial parameter in assessing the clinical status of mechanically ventilated patients in the ICU, directly influencing mortality rates. PO2 measures the amount of oxygen gas dissolved in the blood and is a key indicator of how effectively oxygen is being delivered to the tissues throughout the body. In mechanically ventilated patients, optimal management of oxygenation is vital. Low PO2 levels (hypoxemia) can lead to insufficient oxygen reaching the body's tissues, which is particularly detrimental to vital organs like the brain and heart. Persistent hypoxemia is associated with increased ICU mortality rates because it signifies severe respiratory or circulatory impairment and can lead to multiple organ dysfunction or failure. Conversely, excessively high PO2 levels (hyperoxia) can also be harmful. Over-oxygenation can lead to oxygen toxicity, characterized by damage to lung tissue and other organs due to the generation of reactive oxygen species, which can exacerbate lung injury and also increase mortality. Thus, maintaining a balance in PO2 levels is crucial. Targeted oxygen therapy, aimed at keeping PO2 within a physiologically optimal range, is essential for improving outcomes in mechanically ventilated ICU patients. Management strategies that carefully monitor and adjust oxygen levels can significantly influence survival rates, emphasizing the critical role of precise ventilatory support in critical care setting.",
"**FiO2:** Fraction of inspired oxygen (FiO2) refers to the concentration of oxygen a patient receives during mechanical ventilation and is a crucial factor influencing ICU mortality rates in these patients. FiO2 is adjusted to ensure adequate oxygenation, with normal atmospheric air containing about 21% oxygen, and higher percentages used for medical purposes depending on the patient's needs. Excessively high FiO2 levels, especially sustained over long periods, can be harmful due to the risk of oxygen toxicity. Prolonged exposure to high oxygen concentrations can damage lung tissues through the production of reactive oxygen species, leading to forms of ventilator-induced lung injury. This can worsen patient outcomes and increase ICU mortality rates. Conversely, insufficient FiO2 levels can lead to hypoxemia (low arterial oxygen levels), which jeopardizes oxygen delivery to vital organs and tissues. Chronic hypoxemia can result in organ dysfunction and failure, significantly increasing the risk of death in critically ill patients. Optimal management of FiO2 is therefore critical in mechanical ventilation. Adjusting FiO2 to maintain arterial oxygen levels within a target range minimizes the risk of both hypoxemia and hyperoxia, thereby potentially reducing ICU mortality rates and improving patient outcomes.",
"**Albumin:** Albumin, a major protein in human blood, plays a critical role in maintaining oncotic pressure and transporting substances like drugs, hormones, and enzymes. In the context of ICU patients, particularly those receiving mechanical ventilation, serum albumin levels are an important prognostic indicator. Low albumin levels in ICU patients are often associated with increased mortality rates. Hypoalbuminemia (low albumin concentration) can indicate severe illness or malnutrition, both of which are common in critically ill patients. Low albumin levels compromise the body's physiological response to stress and infection by impairing fluid balance, reducing drug-binding capacity, and diminishing antioxidant defenses. This can lead to a higher susceptibility to complications such as edema, respiratory failure, and sepsis—all of which can exacerbate an already critical condition. Conversely, normal or near-normal albumin levels generally indicate better overall physiological reserves and a potentially lower risk of adverse outcomes. Although the infusion of albumin in some critical care scenarios can help to restore vascular volume and improve organ function, its impact on mortality is debated and appears context-dependent. Thus, monitoring and potentially correcting hypoalbuminemia are considered integral parts of managing critically ill, mechanically ventilated patients, with a direct bearing on improving survival chances in the ICU.",
"**Bicarbonate:** Bicarbonate levels in the blood are a significant indicator of acid-base balance and play a crucial role in the outcomes of ICU patients, particularly those who are mechanically ventilated. Bicarbonate helps to buffer excess hydrogen ions, maintaining pH within a narrow, physiologically optimal range. In mechanically ventilated patients, disturbances in bicarbonate levels can indicate underlying metabolic derangements such as metabolic acidosis or alkalosis, which are often linked with critical illnesses. Low bicarbonate levels often suggest metabolic acidosis, possibly due to renal failure, increased metabolic production of acid, or excessive acid intake. This condition is associated with increased mortality rates in the ICU because it can lead to cardiovascular instability, impaired oxygen utilization by cells, and decreased response to catecholamines. Conversely, elevated bicarbonate levels, which may occur in response to compensatory mechanisms for respiratory acidosis or due to excessive bicarbonate administration, can lead to metabolic alkalosis. This condition can impair oxygen release from hemoglobin due to leftward shifts in the oxygen-hemoglobin dissociation curve and can also cause electrolyte imbalances, particularly hypokalemia, complicating the clinical management of ventilated patients. Effective management of bicarbonate levels, aiming to restore and maintain acid-base balance, is critical in the care of mechanically ventilated patients and can significantly influence ICU mortality rates.",
"**Bilirubin:** Bilirubin, a byproduct of hemoglobin breakdown, is an important biomarker in ICU settings, particularly for patients receiving mechanical ventilation. Elevated bilirubin levels can indicate liver dysfunction or hemolysis and are associated

with increased ICU mortality rates. In critically ill patients, high serum bilirubin often points to hepatic impairment or failure, conditions that are frequently seen in sepsis or as a result of hypoxic or toxic injury to the liver. Liver dysfunction compromises the body's ability to detoxify substances, synthesize critical proteins, and manage infections—all vital functions during critical illnesses. Furthermore, elevated bilirubin levels are a component of the multiple organ dysfunction syndrome (MODS), which is a significant predictor of poor outcomes in the ICU. Increased bilirubin in this context may reflect not just liver problems but also poor overall patient health and the failure of multiple organ systems. Management strategies in the ICU often include monitoring bilirubin levels as part of assessing liver function and overall organ health. Elevated bilirubin can necessitate interventions ranging from supportive care to address the underlying cause (like infection or circulatory failure) to more specific treatments aimed at supporting liver function and preventing further organ damage. Thus, bilirubin levels are a critical prognostic tool in mechanically ventilated patients, with higher levels indicating greater severity of illness and correlating with increased mortality rates.",
"**Creatinine:** Creatinine levels in the blood are a critical marker of kidney function, particularly relevant in the ICU setting for patients undergoing mechanical ventilation. Elevated creatinine levels often indicate acute kidney injury (AKI), which is associated with increased mortality rates in critically ill patients. Acute kidney injury complicates the clinical management of ICU patients because it affects the body's ability to regulate fluid and electrolyte balances, remove waste products, and maintain acid-base balance. AKI often occurs in conjunction with other organ failures, making it part of the broader condition of multiple organ dysfunction syndrome (MODS), which significantly worsens patient prognosis. Elevated creatinine in mechanically ventilated patients can result from a variety of causes, including hypoperfusion due to shock, sepsis, certain medications, or underlying chronic kidney disease. These factors compound the complexity of managing such patients because they require careful fluid management, potential dialysis, and adjustments in medication dosing. The relationship between high creatinine levels and ICU mortality is strong; as creatinine increases, indicating worsening renal function, the risk of death significantly rises. Managing AKI and preventing further kidney damage is a critical component of improving survival outcomes for mechanically ventilated patients in the ICU.",
"**Hematocrit:** Hematocrit, the proportion of red blood cells in blood, significantly impacts ICU mortality rates in mechanically ventilated patients. It serves as a key indicator of oxygen-carrying capacity, fluid status, and overall blood health. In the ICU, both low and high hematocrit levels can be problematic for mechanically ventilated patients. Low hematocrit (anemia) reduces the blood's ability to carry oxygen, compromising oxygen delivery to tissues at a time when demand is often elevated due to critical illness. Anemia in ICU patients can result from chronic disease, acute blood loss, or bone marrow suppression due to sepsis or drug effects. Persistently low hematocrit levels are associated with increased mortality because they can exacerbate the severity of any existing organ dysfunction and may require blood transfusions, which themselves carry risks of infection and immune reaction. High hematocrit levels, on the other hand, indicate hemoconcentration, which can be due to dehydration or polycythemia. Hemoconcentration increases blood viscosity, potentially leading to impaired circulation and increased risk of thrombosis. This condition can compromise microvascular blood flow, particularly in the pulmonary and systemic vascular beds, critical areas for patients on mechanical ventilation. Optimal management of hematocrit levels in mechanically ventilated patients is crucial. Maintaining hematocrit within a target range can improve oxygen delivery and reduce the risk of complications, thus potentially decreasing ICU mortality rates.",
"**Hemoglobin:** Hemoglobin, a critical component of red blood cells, plays a vital role in transporting oxygen from the lungs to tissues throughout the body. In the ICU setting, particularly for mechanically ventilated patients, hemoglobin levels are closely monitored because of their significant impact on patient outcomes. Low hemoglobin levels, or anemia, are common in critically ill patients and are associated with increased mortality rates. Anemia in the ICU can result from a variety of causes including chronic disease, nutritional deficiencies, frequent blood sampling, gastrointestinal bleeding, or bone marrow suppression due to medication or infection. The reduced oxygen-carrying capacity of the blood in anemic patients can exacerbate the severity of any existing organ dysfunction and limit the efficacy of oxygen delivery by mechanical ventilation. This condition may necessitate blood transfusions, which, while potentially life-saving, carry risks of allergic reactions, infections, and lung injury. Conversely, abnormally high hemoglobin levels, although less common, can lead to increased blood viscosity, potentially causing poor microcirculation and an elevated risk of thrombosis, particularly in the pulmonary circulation which is crucial in ventilated patients. Therefore, maintaining hemoglobin levels within an optimal range is crucial in the management of ICU patients. Doing so improves tissue oxygenation and reduces the risk of complications related to both anemia and hemoconcentration, thereby potentially lowering ICU mortality rates.",
"**Lactate:** Lactate levels are a critical biomarker in the ICU, particularly for patients undergoing mechanical ventilation. Elevated lactate levels often indicate tissue hypoxia and metabolic stress, typically associated with increased mortality rates in critically ill patients. Lactate is a byproduct of anaerobic metabolism, which occurs when oxygen delivery to tissues is insufficient to meet metabolic demands. This condition can be due to severe illnesses such as sepsis, shock, or major organ failures that are common in the ICU. Elevated lactate levels signal that the body's tissues are not receiving enough oxygen, which can lead to cell dysfunction and death if not corrected promptly. High lactate levels are a marker of the severity of

illness and are closely linked to outcomes; higher levels are generally associated with poorer prognosis and increased risk of death. Monitoring lactate is vital in the management of mechanically ventilated patients as it helps guide therapeutic interventions aimed at improving tissue oxygenation and overall perfusion. Efforts to reduce lactate levels involve enhancing oxygen delivery through adequate ventilation, optimizing hemodynamics via fluid management and vasoactive medications, and treating underlying causes of increased lactate production. Successfully lowering lactate levels can be indicative of improved cellular function and is associated with better survival rates in the ICU.",

"**Platelet:** Platelet counts are a crucial component in the assessment of critically ill patients in the ICU, particularly those who are mechanically ventilated. The impact of platelet levels on ICU mortality rates can be significant, as abnormalities in platelet count (either thrombocytopenia or thrombocytosis) are associated with various complications and outcomes. Thrombocytopenia, or low platelet count, is more commonly observed in the ICU and is associated with increased mortality rates. This condition can arise from a variety of causes including sepsis, disseminated intravascular coagulation (DIC), major surgery, drug-induced suppression, or bone marrow pathology. Low platelets increase the risk of bleeding, complicate surgical or invasive procedures, and can indicate severe systemic illness, all of which contribute to poorer outcomes. Conversely, high platelet counts (thrombocytosis) can also occur, typically as a reactive process to inflammation, infection, or after splenectomy. While less directly linked to mortality than thrombocytopenia, thrombocytosis can increase the risk of thrombotic events, potentially complicating patient management and recovery. Monitoring and managing platelet counts in ICU patients is essential. Addressing the underlying causes of abnormal platelet counts, whether low or high, can mitigate risks and improve outcomes, particularly in the management of patients requiring mechanical ventilation.",

"**PTT:** Partial thromboplastin time (PTT) is a critical laboratory test used to assess the intrinsic pathway of coagulation in the body. In the ICU, particularly for mechanically ventilated patients, monitoring PTT is vital as it provides insight into the coagulation status and guides therapeutic interventions, especially when anticoagulant therapy is involved. Elevated PTT values in ICU patients can indicate the presence of coagulation disorders such as disseminated intravascular coagulation (DIC), liver disease, or the effects of anticoagulant drugs like heparin. Extended PTT times are associated with an increased risk of bleeding, which can significantly impact mortality rates in critically ill patients. Bleeding complications can exacerbate already severe clinical situations, leading to further deterioration and higher rates of mortality. Conversely, a normal or slightly elevated PTT might be maintained intentionally in patients who are at risk of thrombotic events and are receiving anticoagulation therapy. Managing these levels precisely is crucial to preventing both bleeding complications and thrombosis, which can also be fatal. Therefore, maintaining PTT within a target range is crucial for optimizing patient outcomes. Proper management of anticoagulation and monitoring of PTT can help mitigate the risks of both bleeding and clotting, thereby potentially reducing ICU mortality rates in mechanically ventilated patients.",

"**INR:** The International Normalized Ratio (INR), derived from prothrombin time (PT), is an essential measurement used to assess the extrinsic pathway of blood coagulation. INR is particularly significant in the ICU for mechanically ventilated patients, as it helps monitor and manage clotting risks, guiding anticoagulant therapy. Elevated INR levels in the ICU suggest a higher risk of bleeding due to anticoagulant therapy, liver dysfunction, vitamin K deficiency, or the presence of coagulation disorders such as disseminated intravascular coagulation (DIC). High INR values are associated with increased ICU mortality rates, primarily due to the complications arising from bleeding events. These events can lead to severe outcomes, including hypovolemic shock, anemia, and further deterioration in critical patients. Conversely, a low INR in patients who require anticoagulation (for conditions like atrial fibrillation or mechanical heart valves) indicates insufficient anticoagulation, raising the risk of thrombotic events such as deep vein thrombosis (DVT) or pulmonary embolism, which can also be fatal. Therefore, maintaining an optimal INR range is crucial for mechanically ventilated patients. It balances the risks between thrombosis and hemorrhage, directly impacting the management strategies in the ICU and influencing mortality outcomes. Monitoring and adjusting anticoagulant dosages to achieve and maintain the target INR range can significantly reduce the risk of both bleeding and clotting complications, thus potentially lowering ICU mortality rates.",

"**BUN:** Blood urea nitrogen (BUN) levels are an important clinical marker in the ICU, especially for patients undergoing mechanical ventilation. Elevated BUN levels are often indicative of impaired kidney function or severe catabolic states, and they can significantly influence ICU mortality rates. High BUN levels in the ICU can result from acute kidney injury (AKI), chronic kidney disease, shock, dehydration, or high protein catabolism due to critical illness. These conditions are prevalent among critically ill patients, especially those requiring mechanical ventilation due to the complex interplay of factors like hypoperfusion, sepsis, and multiorgan dysfunction. Elevated BUN levels reflect the severity of these underlying conditions and are associated with poorer outcomes. Increased BUN is linked to higher mortality rates because it often signals renal impairment or failure, which complicates the clinical management of patients. It affects fluid and electrolyte balance, acid-base status, and the clearance of metabolic wastes and medications. Managing these patients requires meticulous attention to fluid management, dialysis if necessary, and careful adjustment of drug dosages to avoid toxicity. Thus, monitoring BUN levels provides crucial information on renal function and the overall metabolic state of mechanically ventilated patients, directly influencing treatment decisions and impacting ICU mortality rates.",

"**WBC:** White blood cell (WBC) count is a critical parameter for assessing the health and immune response of ICU patients, especially those who are mechanically ventilated. Changes in WBC count can significantly influence ICU mortality rates by indicating underlying infection, inflammation, or immune dysregulation. Elevated WBC counts, or leukocytosis, commonly occur in response to infection, stress, or inflammation, such as from sepsis or acute respiratory distress syndrome (ARDS), both prevalent in mechanically ventilated patients. While a high WBC count is a protective and natural response to infection, it can also indicate severe systemic inflammation which, if uncontrolled, leads to tissue damage and organ dysfunction, increasing ICU mortality rates. Conversely, low WBC counts, or leukopenia, can be equally concerning, as they may signify bone marrow suppression, overwhelming infection, or the effects of certain medications. Leukopenia in ICU patients compromises their ability to fight infections, making them more susceptible to acquiring new infections and experiencing worse outcomes. Therefore, monitoring WBC counts in mechanically ventilated patients is crucial. It helps clinicians identify and respond to changes in infection or inflammation status, guiding the adjustment of treatment plans, such as the initiation or alteration of antibiotic therapy, which can directly impact patient survival rates in the ICU.",
"**PaO2/FiO2:** The PaO2/FiO2 ratio, also known as the P/F ratio, is an essential indicator of pulmonary oxygenation efficiency in ICU patients, particularly those on mechanical ventilation. It reflects the ability of the lungs to oxygenate the blood relative to the fraction of inspired oxygen (FiO2) being supplied. This ratio is critical for diagnosing and monitoring the severity of acute respiratory distress syndrome (ARDS) and other forms of respiratory failure. A low P/F ratio indicates impaired gas exchange, suggesting more severe lung damage and possibly a higher risk of mortality. Specifically, a P/F ratio below 300 suggests some degree of acute lung injury, while a ratio below 200 indicates more severe ARDS. Lower P/F ratios are associated with increased mortality rates in the ICU because they reflect more severe lung dysfunction, which is a critical determinant of outcomes in patients requiring mechanical ventilation. Effective management of these patients often involves strategies to improve oxygenation and reduce lung injury, including optimizing ventilator settings, using prone positioning, and considering advanced interventions like extracorporeal membrane oxygenation (ECMO) if necessary. Overall, the PaO2/FiO2 ratio is a valuable prognostic tool in the ICU. It helps clinicians assess the severity of respiratory failure, tailor therapeutic interventions, and gauge the likelihood of survival in mechanically ventilated patients.",
"**BUN/Albumin ratio:** The Blood Urea Nitrogen (BUN)/Albumin ratio is an important marker used in the assessment of critically ill patients, particularly those who are mechanically ventilated in the ICU. This ratio helps evaluate the nutritional status and renal function of patients, and it has implications for their overall prognosis and mortality rates. A high BUN/Albumin ratio typically indicates a combination of renal stress or dysfunction and poor nutritional status. Elevated BUN levels can occur due to acute or chronic kidney injury, which is common in critically ill patients due to factors like hypoperfusion, sepsis, or nephrotoxic drugs. Low albumin levels may reflect malnutrition, liver dysfunction, or a systemic inflammatory response, all of which are prevalent in severe illnesses. This combined indicator is particularly useful in the ICU setting because it correlates with higher catabolic states and more severe systemic illness. Patients with higher BUN/Albumin ratios generally have worse outcomes, including higher mortality rates. This is due to the associated risk of renal failure, complications from malnutrition (such as impaired immune function and wound healing), and the greater likelihood of developing multiorgan dysfunction. Thus, monitoring the BUN/Albumin ratio can provide critical insights into the severity of illness and guide interventions to improve renal function and nutritional support, potentially influencing the survival rates of mechanically ventilated patients in the ICU.",
"**BUN/Creatinine ratio:** The Blood Urea Nitrogen (BUN)/Creatinine ratio is a clinically significant marker often used to assess renal function and hydration status in ICU patients, including those who are mechanically ventilated. This ratio can provide insights into the underlying causes of renal impairment and has implications for patient mortality rates. A normal BUN/Creatinine ratio ranges from approximately 10:1 to 20:1. Deviations from this range can indicate specific health issues. A higher BUN/Creatinine ratio, typically over 20:1, often suggests prerenal azotemia, which can occur due to decreased renal perfusion caused by dehydration, heart failure, or shock. This condition is critical as it may lead to acute kidney injury if not promptly addressed, increasing the risk of mortality in critically ill patients. Conversely, a low BUN/Creatinine ratio, closer to 10:1 or less, may indicate intrinsic renal damage or severe liver disease, reflecting more direct kidney damage or reduced BUN production, respectively. This situation is also associated with higher mortality rates because it often signifies more severe underlying organ dysfunction. Therefore, monitoring the BUN/Creatinine ratio helps in assessing the etiology of renal dysfunction and guiding the management of fluid therapy and cardiovascular support, which are vital in reducing mortality risks in the ICU setting.",
"**Chronic heart failure:** Chronic heart failure (CHF) significantly influences ICU mortality rates in patients who require mechanical ventilation. CHF is a debilitating condition where the heart's ability to pump effectively is compromised, leading to reduced cardiac output and poor systemic perfusion. This underlying cardiac dysfunction exacerbates the challenges faced during critical illnesses, particularly when mechanical ventilation is necessary. Patients with CHF are at a higher risk of developing complications such as pulmonary edema, arrhythmias, and acute on chronic cardiac decompensation when subjected to the stress of critical illness and mechanical ventilation. The strain on the heart from increased respiratory pressures and the metabolic demands of critical illness can lead to further cardiac compromise. Moreover, CHF is often accompanied by

other comorbid conditions like renal impairment, diabetes, and hypertension, which complicate the clinical management and contribute to higher ICU mortality rates. The presence of CHF limits the therapeutic options available for managing fluid balance and cardiovascular stability, critical factors in the care of mechanically ventilated patients. Effective management of these patients involves meticulous monitoring and adjustment of ventilator settings, careful fluid and medication management, and close monitoring of cardiac and hemodynamic status. Despite these efforts, the presence of CHF remains a significant predictor of increased ICU mortality due to the complexity and severity of the underlying disease.",

"**Chronic renal failure:** Chronic renal failure (CRF) significantly impacts ICU mortality rates in patients who are mechanically ventilated, largely due to the compromised physiological reserves and the increased vulnerability to acute complications associated with reduced kidney function. Chronic renal failure refers to the gradual loss of kidney function, which affects the kidneys' ability to filter waste, balance electrolytes, and manage fluid levels—functions that are critical in critically ill patients. In the ICU setting, patients with chronic renal failure often face enhanced risks due to their inability to effectively handle the metabolic and fluid challenges that arise during critical illnesses. These patients are more susceptible to fluid overload, electrolyte imbalances, and acid-base disorders. Such complications can exacerbate existing conditions, leading to further physiological stress and potentially contributing to multiorgan dysfunction. Furthermore, CRF limits the use of certain medications due to decreased renal clearance, complicating the management of these patients. The presence of CRF also often indicates the existence of other comorbidities, such as hypertension and diabetes, which themselves are risk factors for increased ICU mortality. The combination of these factors means that mechanically ventilated patients with chronic renal failure generally have higher mortality rates. Managing these patients requires careful adjustment of fluid therapy, vigilant monitoring of electrolyte and acid-base status, and tailored pharmacological interventions, all aimed at stabilizing their condition and improving outcomes.",

"**Immune suppression:** Immune suppression significantly influences ICU mortality rates, particularly in patients requiring mechanical ventilation. Immunosuppression may be due to chronic conditions like HIV/AIDS, cancer, or autoimmune diseases, or it may be induced by treatments such as chemotherapy, corticosteroids, or other immunosuppressive medications. Immunosuppressed patients are highly vulnerable to infections, including opportunistic infections that typically do not cause disease in individuals with normal immune function. In the ICU, these patients are at an increased risk of developing severe, life-threatening infections such as pneumonia, sepsis, and other nosocomial infections, which are critical complications that can deteriorate their already fragile health status. Furthermore, their capacity to mount a normal inflammatory response to infection is compromised, which can delay the diagnosis and treatment of infections because the usual signs of infection might be attenuated or absent. This delay can lead to a progression of the disease state, complicating the clinical course and potentially increasing mortality rates. Management of immunosuppressed patients in the ICU requires stringent infection control practices, vigilant monitoring for signs of infection, and prompt, aggressive treatment when infections are suspected or confirmed. The need for mechanical ventilation in these patients is often a marker of severe underlying or acute illness, correlating with poorer outcomes and higher mortality rates in this population.",

"**Hypertension:** Hypertension, or high blood pressure, impacts ICU mortality rates for patients who are mechanically ventilated, influencing outcomes through various mechanisms. As a prevalent chronic condition, hypertension often coexists with other serious health issues such as cardiovascular disease, chronic kidney disease, and diabetes, which can complicate the management of critically ill patients. In the ICU setting, patients with a history of hypertension may have altered cardiovascular responses to stress and illness. Chronic hypertension leads to structural and functional changes in the heart and blood vessels, such as left ventricular hypertrophy and increased arterial stiffness. These changes can limit the heart's ability to cope with acute fluctuations in blood pressure and volume, which are common in critical illnesses requiring mechanical ventilation. Furthermore, hypertension increases the risk of acute cardiovascular events like myocardial infarction and stroke, particularly under the stress of critical illness. It also complicates the management of fluid and vasopressor therapies, essential aspects of care for mechanically ventilated patients. Managing hypertensive patients in the ICU often requires meticulous monitoring and adjustment of blood pressure medications, careful fluid management, and close observation for potential cardiovascular complications. Despite optimal management, the presence of hypertension can still correlate with higher ICU mortality rates due to its association with multiorgan vulnerability and severe concomitant conditions.",

"**Metastatic cancer:** Metastatic cancer significantly impacts ICU mortality rates in patients requiring mechanical ventilation. The presence of metastatic disease generally indicates an advanced stage of cancer, where tumors have spread beyond the original site to other parts of the body. This extensive disease progression typically reflects a decline in overall health status and limited physiological reserve, making patients particularly vulnerable in critical care settings. Patients with metastatic cancer often have compromised immune systems, either from the cancer itself or from treatments such as chemotherapy, which increases their susceptibility to infections and other complications. These factors contribute to a higher likelihood of severe complications such as sepsis and multiorgan failure, both common reasons for ICU admission and mechanical ventilation in this patient group. The prognosis for mechanically ventilated patients with metastatic cancer is generally poor, as the advanced cancer often limits the effectiveness of interventions aimed at addressing acute complications. The focus in the ICU may shift

towards palliative care and symptom management, rather than curative treatment, especially when the underlying cancer is not responsive to treatment. Thus, while critical care can temporarily support vital functions, the underlying advanced disease significantly contributes to higher mortality rates among these patients, underscoring the complex interplay between cancer progression and critical illness management.",

"**Obesity:** Obesity impacts ICU mortality rates in mechanically ventilated patients through a variety of physiological and clinical challenges. Obesity is associated with a higher prevalence of comorbid conditions such as diabetes, hypertension, cardiovascular diseases, and obstructive sleep apnea, which can complicate management in the ICU. Mechanically ventilated obese patients face specific challenges related to respiratory mechanics. Obesity, especially abdominal obesity, can impair diaphragmatic movement and reduce lung compliance, increasing the work of breathing and the risk of ventilator-associated lung injury. These respiratory challenges often necessitate adjustments in ventilator settings to ensure adequate ventilation and oxygenation. Despite these complications, studies have shown that obesity paradoxically may be associated with a protective effect in some critically ill patients, a phenomenon known as the obesity paradox. This suggests that obese patients may have better survival outcomes in certain acute medical conditions. However, this effect varies and is not consistently observed across all patient groups or conditions. Furthermore, managing obesity in the ICU requires careful consideration of drug dosing, as obesity affects the pharmacokinetics of medications. Nutritional support must also be meticulously managed to balance energy requirements and avoid further complications. Overall, while obesity introduces significant challenges in the management of mechanically ventilated patients, the relationship between obesity and ICU mortality is complex and influenced by an interplay of factors including the severity of obesity, associated comorbidities, and specific patient conditions.",

"**Diabetes:** Diabetes significantly influences the ICU mortality rates of mechanically ventilated patients through various mechanisms related to its chronic complications and the management challenges it poses. Diabetic patients are more susceptible to infections, particularly bacterial and fungal, which can complicate their ICU course. Moreover, diabetes often coexists with other serious conditions such as cardiovascular disease, chronic kidney disease, and peripheral vascular disease, increasing the overall morbidity and risk of mortality. In the ICU setting, the management of blood glucose levels in diabetic patients is crucial. Hyperglycemia, common in the stress response of critical illness, is linked to worsened outcomes, including an increased risk of infections, prolonged hospital stay, and higher mortality rates. Conversely, aggressive glucose control can lead to hypoglycemia, which is also associated with increased mortality in critically ill patients. Diabetes impacts wound healing and response to infections, further complicating recovery from surgical procedures or acute illnesses that require ICU care. Managing these patients involves meticulous monitoring of glucose levels, careful adjustment of insulin therapy, and vigilant prevention and treatment of possible complications. Thus, diabetes complicates the clinical trajectory of mechanically ventilated ICU patients by exacerbating underlying conditions and complicating metabolic management, thereby affecting overall mortality rates.",

"**Thoracic trauma:** Thoracic trauma significantly affects ICU mortality rates in mechanically ventilated patients due to the critical nature of injuries involved and their impact on respiratory function. Thoracic trauma can include rib fractures, pulmonary contusions, hemothorax, pneumothorax, and cardiac contusions, each potentially compromising respiratory efficiency and cardiovascular stability. Patients with severe thoracic injuries often require mechanical ventilation due to impaired pulmonary function. Rib fractures, for example, can lead to painful breathing, reducing ventilation efficiency and increasing the risk of atelectasis and pneumonia. Pulmonary contusions can result in significant hypoxemia from shunting and decreased lung compliance, further necessitating respiratory support. The presence of thoracic trauma also increases the risk of developing acute respiratory distress syndrome (ARDS), a serious complication characterized by widespread inflammation in the lungs and subsequent acute hypoxemic respiratory failure. ARDS is associated with a high mortality rate, especially when complicated by multiple organ dysfunction syndrome (MODS). Management of thoracic trauma in the ICU requires meticulous ventilatory support to maintain adequate oxygenation and ventilation while avoiding further lung injury. Strategies may include lung-protective ventilation, pain management, and in some cases, surgical interventions to stabilize rib fractures or drain pleural effusions. Overall, thoracic trauma presents significant challenges in the ICU, with the severity of injury and effectiveness of management directly impacting mortality rates in ventilated patients.",

"**Heart rate:** Heart rate is a critical physiological parameter that significantly impacts the ICU mortality rates of mechanically ventilated patients. An abnormal heart rate, whether too high (tachycardia) or too low (bradycardia), can be indicative of underlying health issues or physiological stress, which may compromise patient outcomes. Tachycardia in the ICU can signal a variety of conditions, including fever, pain, hypoxia, hypovolemia, or sepsis. It may also be a response to medications or a manifestation of cardiac issues such as heart failure or ischemia. Persistent tachycardia can increase myocardial oxygen demand and reduce cardiac output, especially if the heart rate is excessively high. This mismatch between oxygen supply and demand can lead to myocardial ischemia and contribute to the deterioration of other organ functions, thereby increasing mortality rates. Bradycardia, on the other hand, may reflect heightened vagal tone, effects of certain medications, or intrinsic cardiac disorders. Severe bradycardia can impair cardiac output and lead to inadequate systemic perfusion, organ hypoxia, and potentially fatal outcomes if not promptly addressed. Management in the ICU involves continuous monitoring and timely intervention to address the causes of abnormal heart rates. Appropriate treatment strategies may include fluid resuscitation, adjustment of ventilatory

parameters, medication review, and, in some cases, the use of pacing or cardioversion techniques. Optimizing heart rate control is crucial for improving survival rates and overall outcomes in mechanically ventilated ICU patients.",
"**Systolic blood pressure:** Systolic blood pressure is a critical indicator of circulatory status and significantly impacts the ICU mortality rates of mechanically ventilated patients. Both abnormally high and low systolic pressures pose serious risks and can influence patient outcomes. Hypotension (low systolic blood pressure) is particularly concerning in the ICU setting, as it may indicate inadequate organ perfusion and hypoperfusion. Common causes in critically ill patients include sepsis, heart failure, and major blood loss. Persistent hypotension can lead to organ dysfunction, including acute renal failure and ischemic injury to the heart and brain, significantly increasing the risk of mortality. Managing hypotension often involves the use of intravenous fluids, vasopressors, and inotropic agents to maintain a target blood pressure and ensure adequate organ perfusion. Hypertension (high systolic blood pressure), while sometimes a physiological response to stress or pain, can also exacerbate cardiac workload and increase the risk of myocardial injury, cerebral hemorrhage, and vascular damage. In mechanically ventilated patients, controlling high blood pressure might require antihypertensive medications, adjustments in ventilatory support, and management of underlying causes such as pain or anxiety. Effective monitoring and management of systolic blood pressure are crucial in mechanically ventilated patients, as maintaining blood pressure within an optimal range can significantly reduce ICU mortality rates and improve overall patient outcomes.",
"**Diastolic blood pressure:** Diastolic blood pressure is a crucial measure in critically ill, mechanically ventilated patients, significantly impacting ICU mortality rates. Diastolic pressure reflects the minimum arterial pressure during the relaxation phase of the cardiac cycle, providing insights into the vascular resistance and overall cardiovascular health of a patient. Low diastolic blood pressure, or diastolic hypotension, can be particularly dangerous in the ICU setting. It may indicate decreased vascular resistance, often associated with conditions like sepsis or medication effects, leading to inadequate coronary artery perfusion. Since coronary arteries are primarily perfused during diastole, excessively low diastolic pressures can result in decreased myocardial oxygen delivery, increasing the risk of cardiac ischemia and other complications that can elevate mortality rates. High diastolic blood pressure suggests increased peripheral resistance, which can strain the heart and exacerbate conditions like left ventricular hypertrophy and heart failure. Persistently high diastolic pressures can also lead to end-organ damage, including renal failure and hypertensive emergencies, thereby contributing to higher mortality rates. Managing diastolic blood pressure involves a balanced approach to ensure that pressures are neither too low to compromise coronary perfusion nor too high to cause end-organ damage. Strategies typically include fluid management, vasopressors, or antihypertensives, tailored to the underlying cause and overall clinical context of the patient. Accurate monitoring and timely intervention for diastolic pressure abnormalities are essential for improving survival outcomes in the ICU.",
"**Mean arterial pressure:** Mean arterial pressure (MAP) is a critical indicator of organ perfusion and is highly influential in determining the ICU mortality rates of mechanically ventilated patients. MAP represents the average pressure in a patient's arteries during one cardiac cycle and is crucial for ensuring adequate blood flow to the organs, especially the kidneys and brain. A low MAP, generally considered below 60 mmHg, can lead to inadequate organ perfusion and hypoxia, which are serious concerns in critically ill patients. Insufficient perfusion can result in acute kidney injury, cerebral ischemia, and other organ dysfunctions, increasing the likelihood of complications and mortality. Management typically involves interventions to raise blood pressure, such as fluid resuscitation and vasopressor administration, aimed at restoring adequate perfusion and preventing organ damage. Conversely, a high MAP can also be problematic, particularly if it reflects increased vascular resistance. This condition can strain the heart and lead to cardiovascular complications, including myocardial ischemia and increased cardiac workload, which can also negatively impact patient outcomes. Maintaining an optimal MAP is thus crucial for critically ill, mechanically ventilated patients. Continuous monitoring and appropriate interventions to manage MAP within a target range are essential strategies to minimize ICU mortality rates, enhancing the chances of survival and recovery.",
"**Respiratory rate:** Respiratory rate is a vital sign that significantly impacts the ICU mortality rates of mechanically ventilated patients. It reflects the patient's ventilatory status and can indicate the severity of their underlying condition and their response to treatment. Elevated respiratory rates in mechanically ventilated patients, often seen with tachypnea, can be indicative of inadequate ventilation, respiratory distress, or a compensatory mechanism for metabolic acidosis. Persistent tachypnea can lead to respiratory muscle fatigue and failure, potentially necessitating increased ventilatory support or higher levels of mechanical ventilation, which are associated with complications such as ventilator-induced lung injury. These conditions can exacerbate patient outcomes and increase mortality rates. Conversely, an abnormally low respiratory rate may signal respiratory depression, potentially caused by sedative or narcotic medications, or it may be a consequence of neurological impairment. Inadequate respiratory rates can lead to hypercapnia (high CO_2 levels in the blood), hypoxia, and acidosis, all of which can also worsen clinical outcomes. Effective management of respiratory rates in mechanically ventilated patients involves careful adjustment of ventilator settings to ensure adequate and efficient gas exchange. Monitoring and adjusting the respiratory rate as needed based on the patient's clinical condition and gas exchange status are crucial for optimizing outcomes and reducing ICU mortality rates.",
"**SpO2:** Oxygen saturation (SpO2) is a crucial parameter for monitoring the oxygenation status of mechanically ventilated

patients in the ICU and significantly impacts their mortality rates. SpO2 measures the percentage of hemoglobin that is saturated with oxygen, providing an immediate assessment of a patient's respiratory efficiency and oxygen delivery to tissues. Low SpO2 levels indicate hypoxemia, which can lead to cellular hypoxia and subsequent organ dysfunction. Persistent or severe hypoxemia is associated with critical outcomes, including increased risk of cardiac arrest, brain damage, and multi-organ failure, all of which can significantly elevate ICU mortality rates. Management typically involves adjusting ventilator settings, such as FiO2 and positive end-expiratory pressure (PEEP), to improve oxygenation while avoiding oxygen toxicity. High SpO2 levels, while generally less concerning, can also be problematic if they result from excessive oxygen administration. Over-supplementation of oxygen can lead to hyperoxia, which may cause oxidative stress and inflammatory lung damage, potentially worsening outcomes in vulnerable patients. Maintaining SpO2 within a target range (typically 92-96% in most ICU settings) is essential. This range optimizes oxygen delivery while minimizing the risks associated with both hypoxemia and hyperoxia, thereby influencing ICU survival rates positively. Effective management of oxygen therapy and careful adjustment of mechanical ventilation are key to achieving these goals.",

"**PEEP:** Positive end-expiratory pressure (PEEP) is a critical setting on mechanical ventilators that significantly impacts ICU mortality rates among mechanically ventilated patients. PEEP works by maintaining a higher pressure in the lungs at the end of exhalation, which prevents alveolar collapse and improves gas exchange by increasing functional residual capacity. Properly adjusted PEEP can substantially benefit patients with acute respiratory distress syndrome (ARDS) or other forms of severe respiratory failure. By stabilizing the alveoli and preventing atelectasis (collapse of part of the lung), PEEP enhances oxygenation and reduces the shunting of blood around oxygen-poor areas of the lung. This improved oxygenation can be crucial in reducing the incidence of hypoxemia-related complications, which are associated with higher mortality rates. However, the application of PEEP must be carefully managed. Excessive PEEP can lead to overdistension of the lungs, increased intrathoracic pressure, and reduced cardiac output, potentially causing hemodynamic instability. This can negate the benefits of improved oxygenation by compromising organ perfusion, particularly affecting the heart and kidneys. Thus, optimizing PEEP levels to balance effective oxygenation while avoiding adverse hemodynamic effects is key. Tailoring PEEP to the individual patient's respiratory mechanics and disease state can significantly influence outcomes, potentially reducing ICU mortality rates in patients with severe respiratory failure.",

"**Temperature:** Temperature regulation plays a critical role in the outcomes of ICU patients, especially those who are mechanically ventilated. Both hyperthermia (high body temperature) and hypothermia (low body temperature) can significantly impact ICU mortality rates by affecting cellular functions, metabolic rate, and susceptibility to infections. Hyperthermia in the ICU can result from infection (such as sepsis), inflammation, drug reactions, or central nervous system dysfunction. Elevated temperatures may enhance immune responses initially but prolonged hyperthermia can lead to cellular damage, increased metabolic demands, and dehydration. If not managed effectively, it can exacerbate existing conditions, leading to worsened outcomes and increased mortality rates. Hypothermia in mechanically ventilated patients is often associated with worse outcomes as well. Low body temperature can impair coagulation cascade, immune function, and metabolic processes, increasing the risk of infections, bleeding, and cardiac events. It can also be indicative of severe sepsis or systemic inflammation, both of which are linked to high mortality rates. Careful management of body temperature, therefore, is crucial. Normothermia (maintaining a normal body temperature) is typically aimed for unless specific therapeutic hypothermia is indicated (e.g., post-cardiac arrest). Active temperature management strategies, including warming blankets, cooling systems, and temperature-adjusted IV fluids, are vital in optimizing patient outcomes and reducing mortality in the ICU.",

"**ARDS:** Acute Respiratory Distress Syndrome (ARDS) is a severe form of respiratory failure characterized by rapid onset of widespread inflammation in the lungs. It is associated with a high mortality rate, especially in the ICU among mechanically ventilated patients. ARDS typically presents with severe hypoxemia and bilateral pulmonary infiltrates, without evidence of cardiac failure or fluid overload. The severity of ARDS is often graded based on the degree of hypoxemia: mild, moderate, or severe, with mortality rates increasing with the severity of the condition. The syndrome can complicate the course of many underlying conditions such as sepsis, trauma, pneumonia, or aspiration, and is a major challenge in critical care due to its complex pathophysiology and management requirements. Management of ARDS involves the use of mechanical ventilation with strategies that aim to minimize further lung injury. These include the use of lower tidal volumes, higher positive end-expiratory pressure (PEEP), and sometimes prone positioning. Despite these interventions, the mortality rate for ARDS remains high, ranging from 35% to 46% depending on severity and underlying health status of the patient. The impact of ARDS on ICU mortality rates is significant due to its complications, including persistent hypoxemia, ventilator-induced lung injury, and the potential for multi-organ failure, emphasizing the importance of optimal management and supportive care strategies.",

"**Organ dysfunction:** Organ dysfunction in mechanically ventilated ICU patients significantly impacts mortality rates, as it often indicates severe systemic disease and a higher risk of multi-organ failure, which is one of the leading causes of death in the ICU. The development of organ dysfunction in critically ill patients can result from a variety of factors, including sepsis, prolonged hypotension, hypoxemia, or direct organ injury. Common forms of organ dysfunction include renal failure, hepatic dysfunction, cardiovascular instability, and respiratory failure. Each type of dysfunction not only complicates the clinical

management of the patient but also interacts synergistically to worsen the patient's overall condition. For example, renal failure can complicate fluid management and electrolyte balance, exacerbating cardiac and pulmonary issues. Similarly, liver dysfunction can affect drug metabolism and coagulation, increasing the risk of bleeding and infection. The presence of any organ dysfunction often necessitates more complex interventions, prolonged mechanical ventilation, and extended ICU stays, all of which can increase the likelihood of adverse outcomes. Statistically, the mortality risk escalates with the number of organs failing. Management strategies focus on supporting failing organs, preventing further damage, and addressing underlying causes. Despite these efforts, organ dysfunction remains a potent predictor of higher ICU mortality rates.",

"**BNP:** B-type Natriuretic Peptide (BNP) levels are a significant biomarker in critically ill, mechanically ventilated patients, impacting ICU mortality rates by indicating cardiac dysfunction. BNP is a hormone produced by the heart's ventricles in response to excessive stretching of heart muscle cells. Elevated levels of BNP in ICU patients typically suggest heart failure or cardiac strain, conditions that can complicate the management of critical illness and affect patient outcomes. High BNP levels in mechanically ventilated patients often correlate with fluid overload, worsening pulmonary edema, and increased cardiovascular stress—all factors that can lead to a higher risk of mortality. The hormone's presence is particularly useful in differentiating cardiac causes of respiratory failure from other etiologies such as pulmonary issues, which is critical in determining the appropriate management strategy. Research shows that elevated BNP levels are associated with increased ICU mortality and morbidity, as they reflect not only current heart dysfunction but also the potential for worsening condition. Monitoring BNP levels can help guide therapeutic decisions, such as the adjustment of fluid management, diuretic therapy, and the use of inotropes, aiming to optimize cardiac output and reduce heart strain. Thus, BNP serves as a crucial prognostic tool in the ICU, aiding in the early identification of cardiac complications among mechanically ventilated patients and influencing interventions that can potentially lower mortality rates.",

"**CPR:** C-Reactive Protein (CRP) is an acute-phase reactant produced by the liver in response to inflammation, and its levels can significantly influence ICU mortality rates in mechanically ventilated patients. Elevated CRP levels are indicative of an active inflammatory response, which may be triggered by infections, autoimmune disorders, or tissue injury. In the context of critically ill patients who require mechanical ventilation, high CRP levels are often associated with adverse outcomes. Elevated CRP can reflect the severity of underlying conditions such as sepsis, pneumonia, or systemic inflammatory response syndrome (SIRS), all of which are common in the ICU and are linked to higher mortality rates. High CRP levels signal an ongoing inflammatory process that, if unchecked, can lead to progressive organ dysfunction and failure. Monitoring CRP levels in ICU patients helps guide clinical management, including the initiation and adjustment of antimicrobial therapy and the evaluation of treatment efficacy. A decreasing trend in CRC levels generally indicates a positive response to treatment, whereas persistently high or rising levels may prompt a reevaluation of therapeutic strategies. Overall, CRP serves as a useful biomarker for assessing the level of systemic inflammation and organ involvement in mechanically ventilated patients, aiding in risk stratification and potentially impacting decisions that affect ICU mortality rates.",

"**PCT:** Procalcitonin (PCT) is a biomarker increasingly used in the ICU to guide the diagnosis and management of bacterial infections and sepsis, which significantly impacts the mortality rates of mechanically ventilated patients. PCT levels rise in response to a systemic bacterial infection, making it a useful tool for distinguishing between bacterial infections and other causes of inflammation. Elevated PCT levels in mechanically ventilated ICU patients typically indicate a severe bacterial infection or sepsis. High levels of PCT have been associated with increased severity of infection and a higher risk of mortality. This is particularly critical in the ICU, where timely and accurate diagnosis of sepsis is essential for initiating appropriate antibiotic therapy and other sepsis management protocols. The use of PCT levels can help in antibiotic stewardship by guiding the initiation and duration of antibiotic treatment. Lowering or normalizing PCT levels often suggest effective control of the infection, potentially leading to a reduced duration of antibiotic use, which is beneficial in preventing antibiotic resistance and reducing side effects. Therefore, monitoring PCT levels in mechanically ventilated patients helps in the early detection and management of bacterial infections, influencing treatment decisions that can directly affect patient outcomes and ICU mortality rates. This targeted approach can improve survival rates by ensuring timely and appropriate treatment of life-threatening infections.",

"**CK:** Creatine Kinase (CK) is an enzyme found predominantly in the heart, brain, and skeletal muscles, and its levels in the bloodstream can significantly impact ICU mortality rates, particularly in mechanically ventilated patients. Elevated CK levels usually indicate muscle damage or stress, which in the context of the ICU can be due to various reasons including myocardial infarction, rhabdomyolysis, trauma, or prolonged immobility. In critically ill, mechanically ventilated patients, high levels of CK may suggest severe muscle breakdown, acute coronary syndromes, or other traumatic injuries, all of which can exacerbate the patient's condition and complicate their recovery. Increased CK in these settings is associated with a higher risk of complications like acute renal failure due to myoglobinuria from muscle breakdown, further increasing mortality risks. Monitoring CK levels helps in the early detection and management of conditions that could lead to increased mortality. For instance, in cases of suspected rhabdomyolysis, early identification and treatment with aggressive fluid resuscitation can help prevent kidney damage and improve outcomes. Similarly, elevated CK levels following suspected cardiac events can trigger

timely interventions that are crucial for patient survival. Therefore, CK serves as a vital biomarker in mechanically ventilated ICU patients, indicating significant muscle damage or stress, which if promptly and adequately managed, can reduce ICU mortality rates.",

"**BE-arterial:** Base excess in arterial blood gas (BE-arterial) is a valuable indicator of the metabolic component of acid-base balance in the body and significantly influences ICU mortality rates in mechanically ventilated patients. Base excess measures the amount of excess or deficit in the base in the blood system, providing insights into metabolic acidosis or alkalosis. A negative base excess (indicating a base deficit) often suggests metabolic acidosis, which can occur due to conditions like renal failure, lactic acidosis, or ketoacidosis. Metabolic acidosis is particularly concerning in critically ill patients because it reflects severe underlying conditions and organ dysfunction. It can compromise cardiovascular stability and reduce the efficacy of catecholamines, potentially leading to worse outcomes and higher mortality rates. Conversely, a positive base excess indicates metabolic alkalosis, which might arise from excessive loss of gastric acid (through vomiting or gastric suction) or overuse of bicarbonates. While metabolic alkalosis is generally less immediately life-threatening than acidosis, it can also lead to complications such as hypokalemia, altered oxygen delivery, and decreased cerebral blood flow, which can negatively impact the patient's condition. Effectively managing base excess through appropriate interventions, such as fluid resuscitation, electrolyte correction, and ventilation adjustments, is crucial in the ICU. Addressing these imbalances promptly helps stabilize critically ill patients, potentially reducing mortality rates among those who are mechanically ventilated.",

"**Lung compliance:** Lung compliance, a measure of the lung's ability to expand and contract in response to changes in pressure, plays a critical role in determining the outcomes of ICU patients, particularly those who are mechanically ventilated. Lung compliance influences how effectively these patients can be ventilated and significantly impacts ICU mortality rates. Low lung compliance, seen in conditions such as acute respiratory distress syndrome (ARDS), pulmonary edema, fibrosis, or pneumonia, makes mechanical ventilation more challenging. Reduced compliance requires higher pressures to achieve adequate ventilation, which can increase the risk of ventilator-induced lung injury (VILI). This injury can lead to barotrauma, volutrauma, and biotrauma, exacerbating lung damage and potentially leading to a cascade of inflammatory responses that can worsen patient outcomes and increase mortality rates. High lung compliance, although less common in the ICU, can also pose challenges, particularly in managing ventilation settings to avoid hyperinflation and oxygen toxicity. Managing patients with altered lung compliance involves careful adjustment of ventilator settings to optimize oxygenation and carbon dioxide removal while minimizing the risk of additional lung injury. Strategies such as lung-protective ventilation protocols, which use lower tidal volumes and appropriate levels of positive end-expiratory pressure (PEEP), are critical. These approaches help to manage lung compliance issues effectively, potentially improving survival rates and reducing complications in mechanically ventilated ICU patients.",

"**AST:** Glutamic oxaloacetic transaminase (GOT), also known as aspartate transaminase (AST), is an enzyme that plays a key role in amino acid metabolism and is found predominantly in the liver, heart, and muscles. Elevated levels of AST in the blood are indicative of cellular damage and are used as a diagnostic marker for liver injury, myocardial infarction, and muscle damage. In the context of ICU patients, particularly those who are mechanically ventilated, high AST levels can significantly impact mortality rates. Elevated AST levels suggest extensive cellular damage which may be due to various critical conditions such as hepatic dysfunction, severe hypoxia, or direct trauma. These conditions often correlate with a more severe clinical course and a higher likelihood of multi-organ dysfunction syndrome (MODS), which significantly increases ICU mortality rates. Monitoring AST levels in ICU patients provides valuable information about the extent of tissue damage and the progression of disease states. Elevated AST levels often necessitate further investigation and intensified monitoring, guiding the management of underlying conditions and therapeutic interventions. Effective management strategies focusing on the causes of elevated AST can mitigate further organ damage and improve outcomes. Therefore, AST is a critical biomarker in assessing the severity and prognosis of critically ill, mechanically ventilated patients, influencing decisions that directly affect ICU mortality rates.",

"**ALT:** Glutamate Pyruvate Transaminase (GPT), also known as Alanine Aminotransferase (ALT), is an enzyme primarily found in the liver and is involved in the metabolism of proteins and amino acids. Elevated levels of ALT in the bloodstream are primarily indicative of liver damage but can also reflect injury to other tissues, including muscle. In the ICU setting, particularly among mechanically ventilated patients, elevated ALT levels often signal acute liver injury or dysfunction, which could be due to factors such as drug toxicity, hypoperfusion, or sepsis. Liver dysfunction is a critical concern as it can compromise the patient's ability to metabolize drugs, produce clotting factors, and maintain glucose and ammonia levels, which are essential for overall metabolic balance. Increased ALT levels in ICU patients are associated with higher mortality rates because they often indicate severe underlying conditions or complications that may lead to multi-organ failure. Monitoring ALT levels can help assess the severity of liver injury and guide management decisions, including adjustments in medication dosages and interventions to support liver function and prevent further damage. Therefore, ALT serves as a significant biomarker in critically ill patients, helping to identify those at increased risk of mortality due to liver-related complications or broader multi-organ dysfunction, thus guiding targeted therapeutic strategies.",

"**LDH:** Lactate Dehydrogenase (LDH) is an enzyme involved in the conversion of lactate to pyruvate in the body's metabolic

process and is present in almost all cells, with particularly high levels found in the heart, liver, muscles, and kidneys. Elevated levels of LDH in the bloodstream are typically indicative of cellular damage and can be used as a marker for tissue breakdown due to various diseases or conditions. In ICU settings, particularly for mechanically ventilated patients, high LDH levels are often associated with several critical conditions including acute respiratory distress syndrome (ARDS), myocardial infarction, liver disease, and severe infections such as sepsis. These conditions can cause extensive cell damage, leading to the release of LDH into the bloodstream. Elevated LDH levels in ICU patients are generally correlated with increased mortality rates. This is because high LDH can reflect more extensive tissue damage and more severe disease states, which complicate patient management and treatment outcomes. The higher the LDH levels, the greater the likelihood of multi-organ failure and the poorer the prognosis. Monitoring LDH levels in mechanically ventilated patients provides valuable diagnostic and prognostic information. It helps in assessing the severity of disease, guiding therapeutic decisions, and evaluating the patient's response to treatment, ultimately influencing ICU mortality rates.",
"**IL-6:** Interleukin-6 (IL-6) is a cytokine involved in the body's inflammatory response and plays a critical role in immune regulation. Elevated levels of IL-6 in ICU patients, particularly those who are mechanically ventilated, often indicate a heightened inflammatory response and are associated with severe infections, sepsis, and other inflammatory conditions. High IL-6 levels are generally linked to worse outcomes and increased ICU mortality rates. This is because IL-6 not only signals the presence of an acute inflammatory state but also correlates with the severity of inflammation and organ dysfunction. Elevated IL-6 levels can drive a systemic inflammatory response that may lead to widespread tissue damage, organ failure, and a condition known as cytokine release syndrome, which is often fatal if not promptly and effectively managed. In mechanically ventilated patients, managing the conditions associated with high IL-6 levels involves using strategies to modulate the immune response and reduce inflammation. This might include the use of corticosteroids, targeted biological agents like tocilizumab (an IL-6 receptor antagonist), and supportive care to maintain organ function. Thus, monitoring IL-6 levels can provide crucial information regarding the inflammatory status of critically ill patients, helping guide therapeutic interventions and potentially impacting ICU mortality rates through timely and targeted treatment.",
"**CVP:** Central Venous Pressure (CVP) is a crucial hemodynamic parameter that reflects the pressure in the thoracic vena cava near the right atrium. It is indicative of the intravascular blood volume status and right heart function. In ICU settings, particularly for mechanically ventilated patients, CVP measurement helps in guiding fluid management and assessing cardiac function, impacting decisions critical to patient outcomes. Elevated CVP can suggest fluid overload, right ventricular dysfunction, or cardiac tamponade, all of which are conditions that can adversely affect the patient's prognosis. High CVP has been associated with increased mortality in critically ill patients, as it often indicates the presence of acute or chronic heart failure and can lead to poor organ perfusion and subsequent organ failure. Conversely, low CVP might indicate hypovolemia, which can also be detrimental if not corrected promptly, leading to decreased cardiac output and potential hypoperfusion of vital organs. Inadequate organ perfusion can result in multi-organ dysfunction and significantly increase the risk of mortality. Thus, maintaining an optimal CVP is essential for mechanically ventilated ICU patients. It ensures adequate fluid balance and cardiovascular function, which are vital for reducing ICU mortality rates. Careful management of fluid therapy, guided by CVP and other hemodynamic parameters, is crucial for optimizing outcomes in these critically ill patients."
